# Octopamine Neuromodulation Regulates Gr32a-Linked Aggression and Courtship Pathways in *Drosophila* Males

**DOI:** 10.1371/journal.pgen.1004356

**Published:** 2014-05-22

**Authors:** Jonathan C. Andrews, María Paz Fernández, Qin Yu, Greg P. Leary, Adelaine K. W. Leung, Michael P. Kavanaugh, Edward A. Kravitz, Sarah J. Certel

**Affiliations:** 1 Center for Structural and Functional Neuroscience, University of Montana, Missoula, Montana, United States of America; 2 Department of Neurobiology, Harvard Medical School, Boston, Massachusetts, United States of America; 3 Division of Biological Sciences, University of Montana, Missoula, Montana, United States of America; Stanford University, United States of America

## Abstract

Chemosensory pheromonal information regulates aggression and reproduction in many species, but how pheromonal signals are transduced to reliably produce behavior is not well understood. Here we demonstrate that the pheromonal signals detected by Gr32a-expressing chemosensory neurons to enhance male aggression are filtered through octopamine (OA, invertebrate equivalent of norepinephrine) neurons. Using behavioral assays, we find males lacking both octopamine and *Gr32a* gustatory receptors exhibit parallel delays in the onset of aggression and reductions in aggression. Physiological and anatomical experiments identify Gr32a to octopamine neuron synaptic and functional connections in the suboesophageal ganglion. Refining the Gr32a-expressing population indicates that mouth Gr32a neurons promote male aggression and form synaptic contacts with OA neurons. By restricting the monoamine neuron target population, we show that three previously identified OA-Fru^M^ neurons involved in behavioral choice are among the Gr32a-OA connections. Our findings demonstrate that octopaminergic neuromodulatory neurons function as early as a second-order step in this chemosensory-driven male social behavior pathway.

## Author Summary

To mate or fight? When meeting other members of their species, male fruit flies must determine whether a second fly is male or female and proceed with the appropriate behavioral patterns. The taste receptor, Gr32a, has been reported to respond to chemical messages (pheromones) that are important for gender recognition, as eliminating Gr32a function impairs both male courtship and aggressive behavior. Here we demonstrate that different subsets of Gr32a-expressing neuron populations mediate these mutually exclusive behaviors and the male Gr32a-mediated behavioral response is amplified through neurons that contain the neuromodulator octopamine (OA, an invertebrate equivalent of norepinephrine). Gr32a-expressing neurons connect functionally and synaptically with distinct OA neurons indicating these amine neurons may function as early as a second-order step in a chemosensory-driven circuit. Our results contribute to understanding how an organism selects an appropriate behavioral response upon receiving external sensory signals.

## Introduction

Organisms live in complicated environments requiring successful interaction with their surroundings for reproduction and survival. Information about the environment is transformed into neural activity by specialized sensory organs that detect signals via touch-, taste-, vibration-, odor- and image-sensitive neurons. Pheromones commonly used as olfactory or contact signals in social behavior like courtship and aggression provide information about gender, receptivity, or conspecificity [1]–[3]. In many systems, chemosensory signal-detecting systems are regulated by biogenic amines including dopamine, serotonin, and norepinephrine (or octopamine, its invertebrate analog) acting as neuromodulators [4]–[6]. Despite extensive investigation in a wide variety of organisms, it has proven difficult to assign specific roles to individual amines in the circuitry concerned with social behavior [7]–[10]. In this study, we directly connect amine regulation to pheromonal communication by identifying specific chemosensory to octopamine neuron contacts and then investigating their tissue-specific functional roles in male aggression and courtship selection.

In *Drosophila*, pheromonal signals are communicated primarily via cuticular hydrocarbons (CHC) and long carbon chain esters that trigger olfactory (volatile) or gustatory (contact) receiving pathways in conspecifics [11]–[13]. Contact pheromones are detected by gustatory receptor-expressing sensory neurons (GRNs) found in taste sensilla in mouth, leg, and wing segments. Despite the importance of this non-volatile sensory information, only a small number of gustatory receptors (GRs) have been reported to be involved in the perception of pheromones that regulate social behavior. In one well-studied example, the behavior of males lacking the gustatory receptor Gr32a is altered in at least three ways; levels of male courtship towards females are reduced, levels of male courtship towards second males are elevated, and aggression as measured by the numbers of lunges (a key higher level behavioral pattern) is reduced [14]–[16]. In addition, a recent study describes a role of tarsal/leg Gr32a-expressing neurons in the inhibition of interspecies courtship between *Drosophila* species [17]. To transduce pheromonal stimuli, axons of Gr32a-expressing neurons project to distinct zones in the suboesophageal ganglion (SOG) [15], [18], and other sites within the central nervous system [19]. The SOG is a central brain region that in addition to axons of gustatory neurons contains extensive neuronal processes of octopamine neurons [20]–[22].

Reduced levels of the amine octopamine (OA) yield phenotypes similar to those seen in flies lacking Gr32a function [23]–[25]. Males without OA exhibit increased male-male courtship [23] and a delay in the initiation of male aggressive behavior [25], as do Gr32a loss-of-function flies [16]. OA function is also necessary for males to make correct choices between courtship and aggression [21], [23] and OA has been suggested to be essential for the display of higher-level aggression [24], [25]. As studies in multiple systems reveal that the context of sensory information and internal states are often shaped molecularly by neuromodulators, we tested the hypothesis that the structural composition of the Gr32a pheromonal network includes synaptic connections to OA neuromodulatory neurons.

We used behavioral assays, Ca^2+^ imaging, and the GRASP (GFP Reconstitution Across Synaptic Partners) method [26], [27] to demonstrate the existence of functional and putative synaptic connections between Gr32a neurons and octopaminergic SOG neurons. Removing Gr32a-expressing neurons, eliminating OA, and altering both simultaneously confirmed essential roles for these chemosensory and OA neuronal groups on male aggression initiation and courtship selection. A role for the labellar Gr32a subpopulation in male aggression was revealed by functionally and anatomically separating Gr32a-expressing neurons into mouth and leg populations. Ca^2+^ imaging experiments demonstrate that OA-expressing neurons in the SOG respond to male cuticular hydrocarbon extracts and this response is eliminated in the absence of Gr32a neurons. Finally, GRASP connectivity between Gr32a neurons and three OA neurons that co-express the male forms of Fruitless (Fru^M^), link anatomical characterization with previous functional data [21] and indicate that this small subset of aminergic neurons is important to provide male selective modulation of behavior. The results presented here begin to decipher social behavior at the level of small subsets of sensory and neuromodulatory neurons and provide insight into how amine-expressing neurons anatomically contribute to circuitry directing sex-specific behavior.

## Results

### Gr32a neurons contact OA neurons in the suboesophageal ganglion

**Figure 1:**
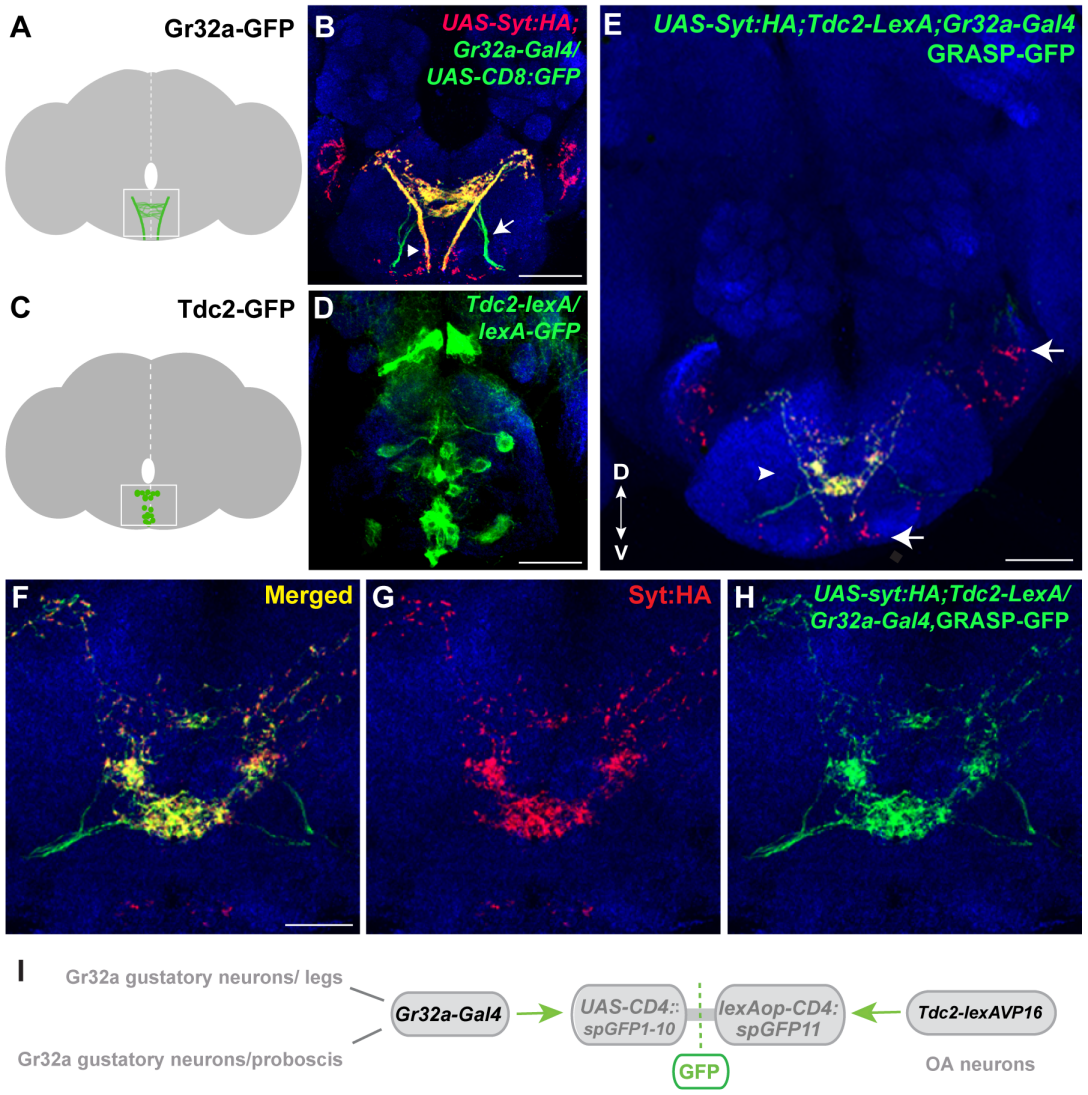
Gr32a neurons contact OA neurons in the suboesophageal ganglion. (**A**) Schematic depicting the SOG region targeted by Gr32a axons visualized in panel B. (**B**) Axons and presynaptic terminals of Gr32a-expressing neurons identified by immunofluorescence to CD8:GFP and the synaptotagmin:HA fusion protein in *UAS-sytHA;;UAS-CD8:GFP/Gr32a-Gal4* progeny (green, anti-CD8, Invitrogen; red, anti-HA, Roche). Sensory neurons from the labellum project through the labial nerve (arrow), mouthpart neurons project through the pharyngeal/accessory nerve, and neurons from thoracic ganglia project via the cervical connective (arrowhead). Scale bar represents 30 µM (**C–D**) A subset of Tdc2-positive neurons located in the SOG in a schematic (C) and with GFP expression driven by the Tdc2-LexA line (*Tdc2-LexA;lexAop-rCD4:GFP*. Cell bodies are visible (D) with extensive arborizations apparent in a series of optical sections ventral to the cell bodies (Figure S1). (**E**) GRASP-mediated GFP reconstitution is observed between Gr32a neurons expressing CD4::spGFP1-10 and *UAS-syt:HA* (red, anti-HA, Roche) and OA neurons expressing CD4::spGFP11. GRASP reconstitution is detected by immunofluorescence using a rabbit monoclonal GFP antibody (Life Technologies). Regions in the SOG with only syt:HA expression are indicated (arrows) in addition to GFP-reconstitution contacts that show co-localization with syt-HA expression (arrowhead). Scale bar is 50 µM. (**F–H**) Optical sections of the same brain at higher magnification showing GRASP-mediated GFP reconstituted expression (H), syt:HA localization (G) and clear overlap or close association at synaptic-like puncta in the merged channel (F). Scale bar represents 20 µM. (See also Figure S2.) (**I**) Schematic representation of the GRASP reporter lines combined with the *Gr32a-Gal4* and *Tdc2-lexA* driver lines.

To test the hypothesis that OA neurons might anatomically function in the Gr32a pheromonal input pathway, we generated a *Tdc2-LexA:VP16* line and utilized this tool with the split-GFP system developed in *C. elegans* [26] and adapted for *Drosophila* [27]. In invertebrates, OA is synthesized from the amino acid tyrosine via the action of tyrosine decarboxylase (TDC) and tyramine β-hydroxylase (Tβh). The *Tdc2* gene encodes the neuronal TDC [28] and the *Tdc2-LexA* line can be used to label and manipulate OA neurons ([29], Figure S1) and possibly a small population of tyramine (TA)-expressing neurons [20]. The Gr32a receptor is expressed in sensory neurons in the mouth (labellum - a gustatory organ of the proboscis and pharynx) and in tarsal segments of all three legs [14], [15], [30]. Axons of Gr32a-expressing neurons project through three peripheral nerves to the SOG (Figure 1A,B) [18], [31]–[33]. Peripheral chemosensory neuron expression of OA has not been detected in this study or previously [28]. However, within the central brain, individual OA neurons project extensive arborizations targeting multiple neuropil regions including the SOG, which functions at least in part, to receive key contact pheromone information (Figure 1C,D, Figure S1D) [20], [21], [28].

To determine if Gr32a-expressing neurons directly contact OA neurons, we used the GFP Reconstitution Across Synaptic Partners (GRASP) method, which detects putative synaptic connections based on the reconstitution of two fragments of a split-GFP protein on the outer membrane of targeted neuronal populations [26], [27]. We observed GFP reconstitution in a reproducible, distinct pattern within the central SOG (Figure 1E–I) in flies containing one fragment of split-GFP under *Tdc2* (OA/Tyramine) control (*Tdc2-lexA; lexAop-CD4::spGFP11*) and the second fragment driven by the promoter of *Gr32a* (*Gr32a-Gal4; UAS-CD4::spGFP1-10*). Little or no fluorescence was observed upon expression of either split-GFP fragment alone (Figure S2).

To confirm that at least a portion of the fluorescence seen in contact zones is likely synaptic, we added the *UAS-syt:HA* reporter [34] (Figure 1E–G, displayed as red puncta). The overall syt:HA pattern shows clear preferential localization of terminal regions of Gr32a neurons and an extensive overlap is seen between syt:HA localization and split-GFP reconstitution at both low and higher magnification (Figure 1E–H). In the merged channels (Figure 1E,F), regions of syt:HA expression where no GFP reconstitution is observed indicating that only specific neurons amongst the populations of Gr32a and OA neurons contact each other. In particular, the synaptic endings derived from Gr32a neurons that project directly to the ventrolateral protocerebrum region [15] do not express reconstituted GFP (Figure 1E, arrow) demonstrating specificity in the GFP reconstitution pattern and specificity in the Gr32a to OA neuronal connections. This anatomical data is consistent with a recent study suggesting a close, possibly synaptic, apposition of Gr32a-expressing axons with male mAL neurons [14].

Gr32a expression is seen in all bitter-sensing neurons within the sensilla of the labellum, usually accompanied by many additional gustatory receptors in most of the neurons [33], [35], [36]. In one subgroup of chemosensory neurons, the Gr22e (9 neurons) and Gr59b (4 neurons) receptors co-localize with Gr32a as has been reported previously [33], while in another distinct group Gr32a and Gr47a co-localize (3 neurons) [36]. Expressing *Gr22e-Gal4* or *Gr59b-Gal4* with *Tdc2-lexA* and the GRASP reporter transgenes resulted in split-GFP reconstitution in the SOG region as described above (Figure 1) albeit with reduced GRASP expression likely due to co-expression in only a subset of the population of Gr32a neurons (Figure S3). We also examined whether OA neurons might receive synaptic input from the Gr47a/Gr32a neurons, a different subgroup of bitter-responsive neurons [31], [37]. GFP reconstitution was not observed between the *Gr47a-Gal4* labeled axons and OA neurons (Figure S4). Although definitive verification of the GRASP signals will require electron microscopy, our results suggest that a number of octopaminergic SOG neurons may serve as neuromodulatory links in the information pathways between specific Gr32a-expressing neurons and taste-related behavioral outputs.

### Removing OA neurons changes Gr32a SOG axonal targeting

**Figure 2:**
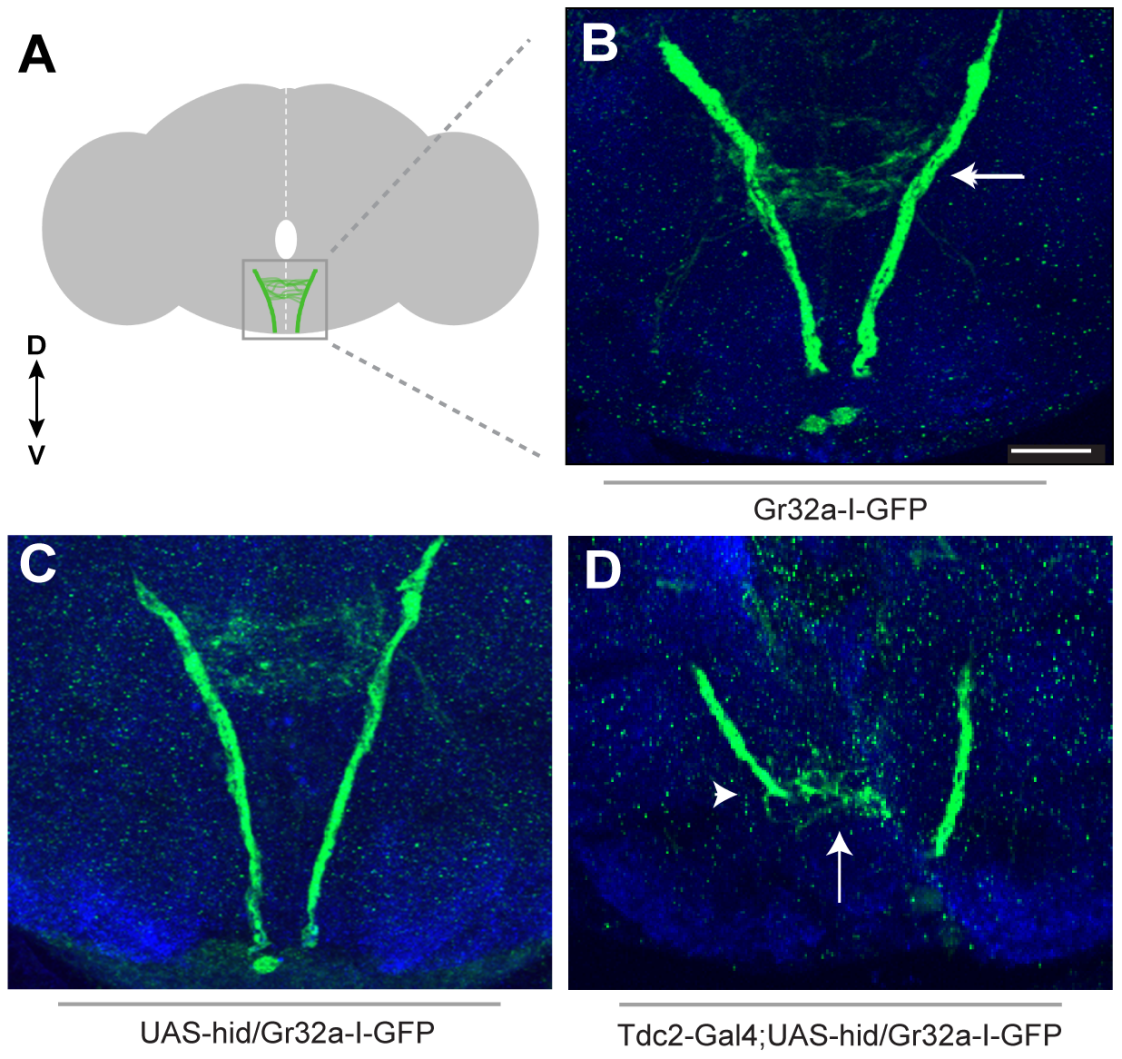
Removing OA neurons significantly alters Gr32a axonal projections. (**A**) Schematic representation of the adult brain with Gr32a-expressing axonal arborizations in the SOG. (**B**) *Gr32a-I-GFP* expression in a typical wildtype adult brain. The Gr32a-expressing neurons located in the tarsi, labellum, and mouthparts all terminate in the SOG (arrow). (**C**) Confocal sections of a *UAS-hid UAS-Red Stinger* control brain verifying wildtype organization of *Gr32a-I-GFP* projections (**D**) Confocal sections of transgenic *Tdc2-Gal4/UAS-hid UAS-Red Stinger;Gr32a-I-GFP* adult brains. When all OA neurons are eliminated, a range of axonal projection defects was observed including a severe reduction and disorganization of Gr32a leg and labellum termini (arrow, arrowhead). Scale bar represents 30 µM.

If a subset of Gr32a gustatory neurons are in synaptic contact with octopaminergic SOG interneurons, then removing the OA neurons might cause changes in the branching patterns of incoming Gr32a axonal projections. To test this hypothesis, we eliminated OA neurons by driving expression of the programmed cell death gene, *head involution defective (hid*, *UAS-hid)*, coupled with the *UAS-Red Stinger* reporter transgene in OA/TA neurons. The *Tdc2-Gal4/UAS-hid UAS-Red Stinger* combination allowed us to identify transgenic brains that retained OA neurons (DsRed expression was observed) and brains that were devoid of OA neurons (DsRed and Tβh expression were absent) (Figure S5). Gr32a neuronal projections entering the SOG were visualized using the Gr32a-I-GFP reporter construct (Figure 2A–C) which drives GFP expression as a direct promoter fusion [33]. The resulting GFP fluorescence is weaker than when amplified through the Gal4/UAS system, however when all OA neurons were eliminated, we observed a range of axonal projection defects including an absence of Gr32a-I-GFP immunoreactivity in the SOG (data not shown, 31%) or a severe reduction and disorganization of Gr32a leg and labellum termini in 69% of preparations (n = 21, Figure 2D). Since the adult brains were dissected 1–5 days after eclosion, the differing severity of the Gr32a projection phenotypes could be due to increased axonal disorganization in the absence of OA neuronal targets as the flies age. No similar disorganization of Gr32a axonal projections is observed in control brains during the 1–5 day time frame.

We next asked if Gr32a axonal morphology is altered if OA neurons are present but lack OA due to a null mutation in *Tyramine β-hydroxylase* (*tβh^nM18^*). Using *Gr32a-Gal4* to drive reporter GFP expression, the stereotypical projections of Gr32a-expressing neurons from control and OA deficient males were examined. Gr32a axons terminated in the SOG (Figure S6) in heterozygous control adult brains (*tβh^nM18^/+;Tdc2-Gal4;20XUAS-6XGFP*). Compiling the same number of confocal sections in controls and OA deficient male brains *(tβh^nM18^;Tdc2-Gal4;20XUAS-6XGFP)* indicates the majority of Gr32a projections reach the SOG as in controls. However, we observed aberrant termination of Gr32a axons in the antennal lobe region of OA deficient brains (Figure S6C–E) that is distinct from previously described projections into the ventrolateral protocerebrum [15]. The effects of eliminating production of OA on individual Gr32a-expressing neurons remains to be determined but results from these experiments suggest the correct differentiation of OA neurons is required for precise axon targeting by at least a subset of Gr32a chemosensory neurons.

### Gr32a expressing neurons mediate onset of aggression via OA signaling

**Figure 3:**
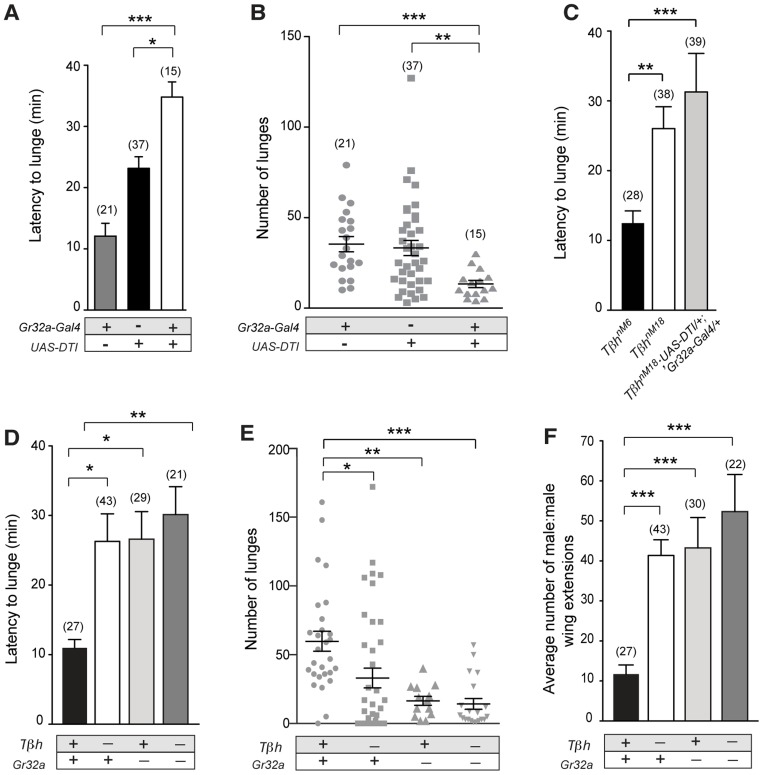
Gr32a-expressing neurons promote aggression via OA signaling. (**A–B**) Fights between males with Gr32a-expressing neurons removed by expressing Diptheria Toxin *(UAS-DTI;Gr32a-Gal4)* and individual transgenic controls, *UAS-DTI* or *Gr32a-Gal4*. (**A**) The latency to first lunge was significantly higher in *UAS-DTI/+*; *Gr32a-Gal4/+* males as compared to controls (all statistical tests are Kruskal-Wallis with Dunn's multiple comparison test except where noted, ***p<0.001, *p<0.05). (**B**) Number of lunges (represented by each dot) performed in a 30 min period after the first lunge by any control or experimental male in a fighting pair. Males without Gr32a neurons exhibited a significant reduction in lunges as compared to controls (***p<0.001, **p<0.01). (**C**) Fights between control male pairs (revertant *tβh^M6^* allele), experimental males without OA (revertant null mutation, *tβh^nM18^*), or experimental males without OA and without Gr32a-expressing neurons (*tβh^nM18^;UAS-DTI/+*; *Gr32a-Gal4/+*). The latency to first lunge was significantly higher in males without OA and in experimental males compared to control males (**p<0.01) and not statistically different between males without OA and experimental *tβh^nM18^;UAS-DTI/+*; *Gr32a-Gal4/+* males. (**D–F**) Fights between control male pairs (revertant *tβh^M6^* allele) and three groups of experimental males; without OA = *tβh^nM18^*, without Gr32a receptors = *tβh^M6^;;Gr32a^−/−^*, and without OA and Gr32a receptors = *tβh^nM18^;;Gr32a^−/−^*). (**D**) The latency to first lunge was significantly higher in males without OA (*tβh^nM18^*) and in experimental males without OA and the Gr32a receptor (*tβh^nM18^; Gr32a^−/−^*) or without only the Gr32 receptor (*tβh^M6^; Gr32a^−/−^*) males as compared to control *tβh^M6^* males (One way ANOVA, *post hoc* Tukey's comparison, *p<0.05, **p<0.01). (**E**) The number of lunges by pairs of experimental males were significantly less than exhibited by control males but not when compared to each other (***p = 0.0002, **p = 0.002, *p = 0.01). (**F**) The average number of wing extensions directed toward the second male in each aggression assay. The number of wing extensions exhibited by males without the Gr32a receptor and without OA, and males without Gr32a receptors were significantly greater than control *tβh^M6^* males (***p<0.001) but not males without OA (*tβh^nM18^*). Error bars denote s.e.m.

A previous study reported that the Gr32a receptor mediates aggression-inducing and courtship suppression effects of the male-enriched cuticular hydrocarbons, (*z*)-7-tricosene [16]. Results presented here indicate that Gr32a-expressing neurons contact OA neurons and suggest that octopaminergic signaling is one of the pathways through which Gr32a-mediated pheromonal information is conveyed to other brain or possibly ventral cord regions. To test this hypothesis, we first analyzed fighting defects in males with impaired Gr32a function in our aggression chambers. This data provides a baseline for calculating how removal of OA neuromodulation in addition to eliminating Gr32a-mediated pheromonal information may or may not further alter male aggression or courtship. We ablated Gr32a-expressing gustatory neurons through expression of Diphtheria Toxin *(UAS-DTI)* via the *Gr32a-Gal4* driver line [38]. Pairs of *UAS-DTI;Gr32a-Gal4* or transgenic control males were placed in an aggression chamber and latency to the first lunge (a key aggressive pattern essential for the establishment of hierarchical relationships) and total numbers of lunges were quantified. Consistent with a role of Gr32a-expressing neurons in perceiving pheromones utilized for sex and species recognition in males, the latency to first lunge was significantly longer in males without Gr32a neurons compared to parental controls (Figure 3A). Moreover, a significant reduction in the number of lunges was also observed (Figure 3B). Males without Gr32a neurons exhibited a reduction in aggressive behavior when paired with a single control male as demonstrated by few lunges per fight and a failure to initiate aggression (Figure S7A–C).

To test the behavioral consequences of removing both Gr32a-expressing neurons and OA, we added the *UAS-DTI;Gr32a-Gal4* transgenes to males with either the *w^+^ tβh^nM18^* null recombinant chromosome) or the *w^+^ tβh^M6^* recombinant control chromosome [23]. The resulting experimental males do not produce OA yet retain OA neurons and the Gr32a-expressing neurons are ablated. Similar to what was observed for flies without Gr32a neurons, flies without OA show a 2-fold increase in latency when compared to genetic control males (Figure 3C). If the function of Gr32a and OA neurons in setting the timely onset of an aggressive response were independent, the absence of both Gr32a receptors and OA function should result in an additive effect on aggression latency as compared to single mutants (flies lacking Gr32a-expressing neurons or OA only). Removing Gr32a signaling and OA via the *tβh^nM18^* mutation did result in a small increase in the latency to the first lunge when compared to control males (Figure 3C). However, the increased latency was not significantly different from that observed in males without OA only (Figure 3C, (Mann-Whitney U test, p = 0.4). This equivalent aggression initiation delay exhibited by males without Gr32a neuronal function and *tβh^nM18^*;*UAS-DTI;Gr32a-Gal4* males is the expected result if the aggression-promoting pheromonal signals transmitted by Gr32a neurons are at least partially conveyed via OA neurons. When males without OA and Gr32a neurons fight, the total lunges per fight are decreased (Figure 3D), though, the reduction in lunge number is not substantially different from *UAS-DTI;Gr32a-Gal4* males (Figure 3B). Removing Gr32a neurons in males without OA significantly decreased lunge number (Figure S7D), however this additive value in lunge number reduction is not observed in males with only the Gr32a receptor eliminated (see below, Figure 3E).

Males with lowered levels of OA have been reported to exhibit lower numbers of lunges [24], [25]. Results in this study indicate that *tβh^nM18^* mutant males take twice as long as controls to display their first lunges in fights (Figure 3C,D, Figure S7E). We previously demonstrated that males without detectable OA exhibited elevated courtship behavior towards other males [23]. One possible explanation of these results is that OA deficient males have difficulty recognizing the sex or species of a second fly. A similar delay in initiation observed in fights between males lacking Gr32a receptor neurons may be for this same reason (this study and [16]). Given such a large delay in the onset of aggression in OA mutant flies (Figure 3C,D and [25]), at least two factors can impact how lunge numbers are counted. First, counting lunges for a set period of time beginning when flies are first introduced to a chamber can yield very different results from counting at the start of lunging behavior (Figure S7E). A second consideration is the inclusion of male pairs that did not display lunges. If fights without lunges are scored as “zeros”, the numbers of lunges seen in fights between pairs of *tβh^nM18^* males are significantly lower than the numbers seen in the genetic controls (Figure S7F), when fights that do not exhibit lunging are excluded, significant differences between *tβh* control and experimental are not found (Figure S7G). *tβh^nM18^* males that exhibited low numbers of lunges also engaged in elevated levels of male-male courtship, which was not observed in *tβh^M6^* controls while OA deficient males that exhibited high numbers of lunges engaged in male-male courtship at low levels. These results are displayed as a ratio of wing extensions (singing) divided by lunges (Figure S7H). Thus the affects of removing OA on the intensity of aggression also include a critical delay in the onset of aggression and an increase in male-male courtship.

To support the hypothesis that Gr32a receptor function itself is a key transducer of the aggression-enhancing stimuli regulated by OA, we tested males containing the *Gr32a^−/−^* mutation [15] in the *tβh^nM18^* (null for OA) and *tβh^M6^* (control) backgrounds. Males without the Gr32a receptor and males without OA and Gr32a exhibited a similar 2-fold increase in the latency to lunge (Figure 3D). The number of lunges displayed by males without OA (*tβh^nM18^*), without Gr32a (*tβh^M6^;;Gr32a^−/−^*), or without OA and the Gr32a receptor (*tβh^nM18^;;Gr32a^−/−^*) were each significantly reduced as compared to control males (*tβh^M6^*) (Figure 3E). Differences in lunge number between groups of experimental males were not observed (Figure 3E) providing further support that OA may be downstream of Gr32a sensory signaling processes.

As separately removing OA and Gr32a receptor function has been reported to increase male-male courtship toward intact males [23] and decapitated males [15], we quantified the occurrences of courtship to the second male within the aggression paradigm. Males without the Gr32a receptor, males without OA, and males without OA and Gr32a all displayed a significantly greater amount of male-male courtship to the second intact male compared to controls (Figure 3F). As with parameters of aggression, removing OA in the context of the *Gr32a^−/−^* mutation does not increase the already elevated levels of male-male courtship suggesting that OA may modulate Gr32a sensory input related to suppressing conspecific male courtship and promoting male aggression as these two processes have been suggested to reflect independent, parallel processes [39].

### The intracellular Ca^2+^ response of OA SOG neurons to male CHCs requires Gr32a neurons

**Figure 4:**
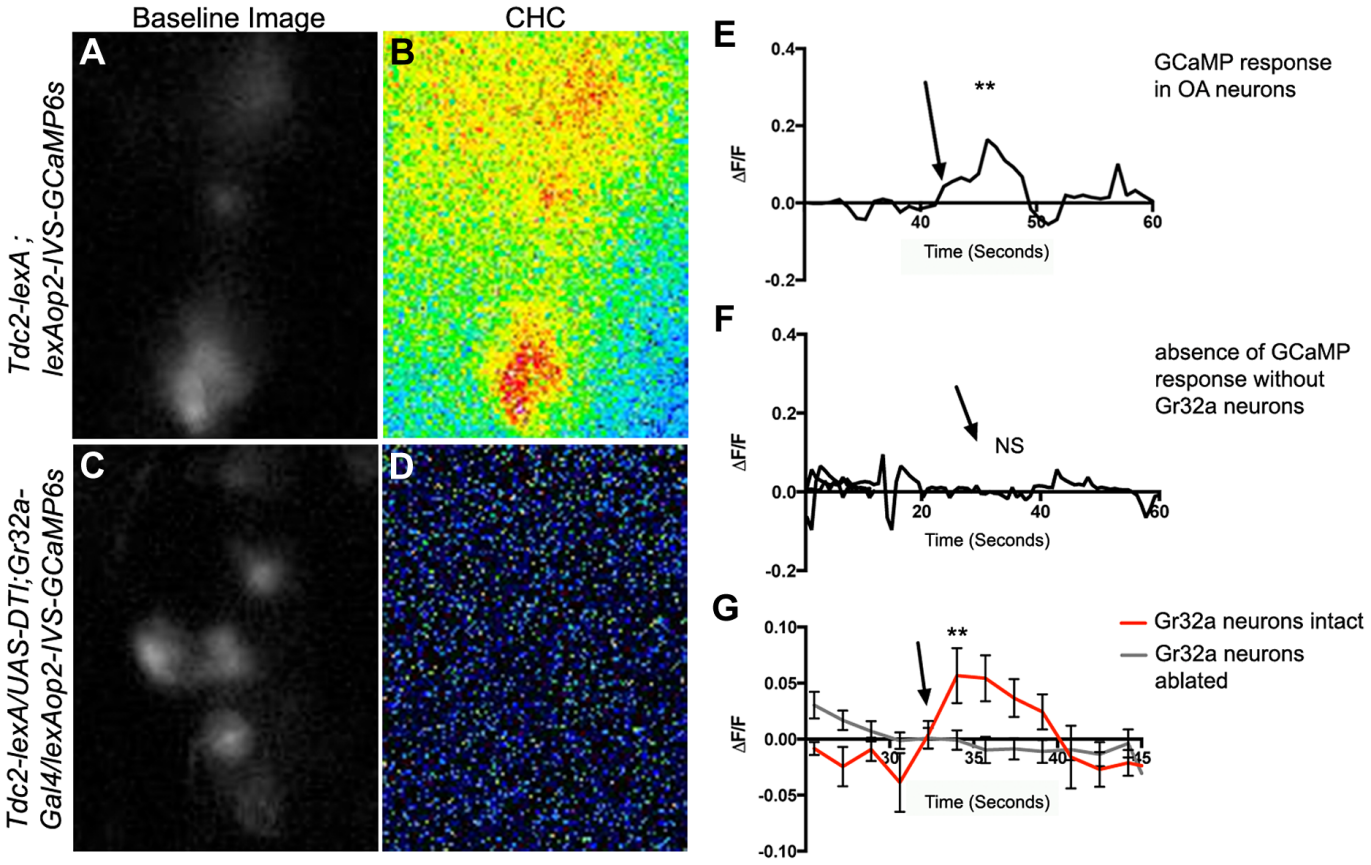
Male CHCs evoke intracellular Ca^2+^ responses in OA neurons that are dependent on Gr32a neurons. (**A**) Greyscale image (background subtracted) of GCaMP3 fluorescence in OA neurons located within the SOG in a *Tdc2-lexA;lexAop2-IVS-GCaMP6s* male. (**B**) Pseudocolored subtraction image demonstrating an increase in fluorescence in response to male CHC application. (**C**) Greyscale image (background subtracted) of baseline fluorescence in the SOG of a male with Gr32a neurons eliminated (*Tdc2-lexA; UAS-DTI;Gr32a-Gal4/LexAop2-IVS-GCaMP6s*). (**D**) No changes in fluorescence are observed in the pseudocolored subtraction image of OA SOG neurons when male CHC extract is administered to the legs of males lacking Gr32a neurons. (**E**) A representative calcium signal trace of OA neurons expressing GCaMP6s in panels A–B in response to male CHC extract application (arrow), unpaired t-test **p<0.006. (**F**) A representative trace demonstrating the lack of calcium response in OA neurons after male CHC extract application (arrow) to the legs of males without Gr32a neurons (*Tdc2-LexA;UAS-DTI;Gr32a-LexA/20XLexAop2-IVS-GCaMP6s*). (G) The average calcium response of eight regions of interest from five *Tdc2-lexA;lexAop2-IVS-GCaMP6s* males (red line) before and after male CHC administration (arrow, unpaired t-test, **p<0.0001). The gray line is the average calcium response of ten regions of interest from five *Tdc2-LexA;UAS-DTI;Gr32a-LexA/20XLexAop2-IVS-GCaMP6s* males in response to male CHC administration. No significant change in response was observed (unpaired t-test, p<0.0788). Error bars denote s.e.m.

To determine if OA-expressing neurons modulate male aggression and courtship behavior by responding to sensory information concerning sexual recognition, we expressed the genetically encoded calcium indicator GCaMP6 [40], and assayed changes in intracellular Ca^2+^ responses evoked by application of CHC extracts to the male legs. Male CHC extracts evoked significant increases in GCaMP6s fluorescence in subsets of OA SOG neurons of *Tdc2-LexA;20XLexAop2-IVS-GCaMP6s* males (Figure 4A–B, E,G, n = 8). The response to male CHCs was abolished in males with Gr32a neurons eliminated via DTI expression (*Tdc2-LexA/UAS-DTI;Gr32a-Gal4/20XLexAop2-IVS-GCaMP6s*) (Figure 4C–D, F,G, n = 10) or through *UAS-hid* expression (*Tdc2-LexA/UAS-hid UAS-RedStinger;Gr32a-Gal4/20XLexAop2-IVS-GCaMP6s*, data not shown). Male CHC extracts were also applied to the forelegs of males expressing GCaMP3.0 in Gr32a neurons *(UAS-GCaMP3.0/Gr32a-Gal4)*, however Ca^2+^ changes were not reliably detected in these foreleg neurons. As the cellular transduction mechanisms involved in Gr32a signaling are currently unknown, it is possible that Ca^2+^ changes may be near or below the detection threshold or that a response may not include a Ca^2+^ influx. Nevertheless, our physiological data support the hypothesis that sensory information received by Gr32a neurons is directly relayed to OA neurons in the SOG.

### Subset-specific effects of Gr32a neuronal function on male aggression and courtship selection

**Figure 5:**
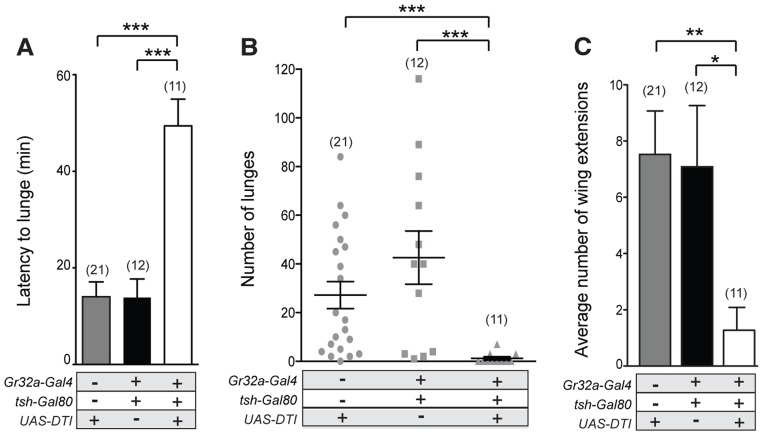
Gr32a chemosensory neurons located in the mouth promote aggression without an elevation in male-male courtship. (**A–B**) Fights between males with the Gr32a-expressing mouth neuronal population removed by expressing Diptheria Toxin *(UAS-DTI)* through the *Gr32a-Gal4* line with Gal4 activity in the legs blocked by *tsh-Gal80*. Separate transgenic controls, *UAS-DTI/+* and *tsh-Gal80/+; Gr32a-Gal4/+* were scored. (**A**) The latency to first lunge was significantly higher in *UAS-DTI/tsh-Gal80*; *Gr32a-Gal4/+* males as compared to controls (Kruskal-Wallis with Dunn's multiple comparison test, ***p<0.001). (**B**) Number of lunges performed per 30 min period after the first lunge by controls or experimental *UAS-DTI/tsh-Gal80*; *Gr32a-Gal4/+* males. Each dot represents the numbers of lunges performed by either male in a fighting pair. Males without Gr32a-expressing mouth neurons exhibited a significant reduction in lunges as compared to controls (Kruskal-Wallis test with Dunn's multiple comparison test, ***p<0.001). (**C**) The average number of wing extensions directed toward the second male in each aggression assay. The number of wing extensions exhibited by males without mouth Gr32a neurons were less than control males (Kruskal-Wallis with Dunn's multiple comparison test, *p<0.05, **p<0.01). Error bars denote s.e.m.

Although a single receptor subtype, Gr32a, appears to mediate key pheromonal responses that inhibit interspecies courtship, promote male aggression, and suppress conspecific male-male courtship, different subpopulations of Gr32a-expressing neurons may be involved in each case. To test this idea, we selectively ablated Gr32a-expressing chemosensory neurons located in the mouth without removing the leg Gr32a neurons. For this purpose, we used the homeotic *teashirt* promoter driving Gal80 expression [41] to significantly block Gal4-mediated activation in regions outside of the head. Via this route Diphtheria Toxin expression *(UAS-DTI)* was prevented resulting in males lacking Gr32a-expressing neurons only in the labellum or mouth (Figure S8). As in experiments presented above, the latency to lunge was significantly longer in males without labellar Gr32a neurons (Figure 5A) and a significant reduction in lunge number was also observed (Figure 5B). As increased male-male courtship to a second intact male is exhibited by males without the Gr32a receptor and without OA (Figure 3F), we quantified the occurrences of courtship behavior (wing extensions and abdomen bending). The male-male courtship levels of *UAS-DTI;teashirt(tsh)-Gal80/Gr32a-Gal4* male pairs are lower than control levels (Figure 5C) yet experimental males court females and successfully copulate in courtship assays (92%, n = 13) albeit with a longer latency to initiate courtship (Table S1). The ability of experimental males to successfully copulate is in agreement with a report indicating the ablation of the entire Gr32a neuron population does not alter the courtship of conspecific females [17]. Our results thereby indicate that there are functional differences on male social behavior served by the two separate populations of Gr32a-expressing chemosensory neurons and that the labellar Gr32a subpopulation is important for male aggression. Experiments in this study do not exclude a role for Gr32a leg neurons in male aggression, however the functional importance of the tarsal Gr32a subpopulation on male interspecies courtship behavior has recently been described [17].

### Tissue-specific refinement of Gr32a to octopamine neuron synaptic contacts

**Figure 6:**
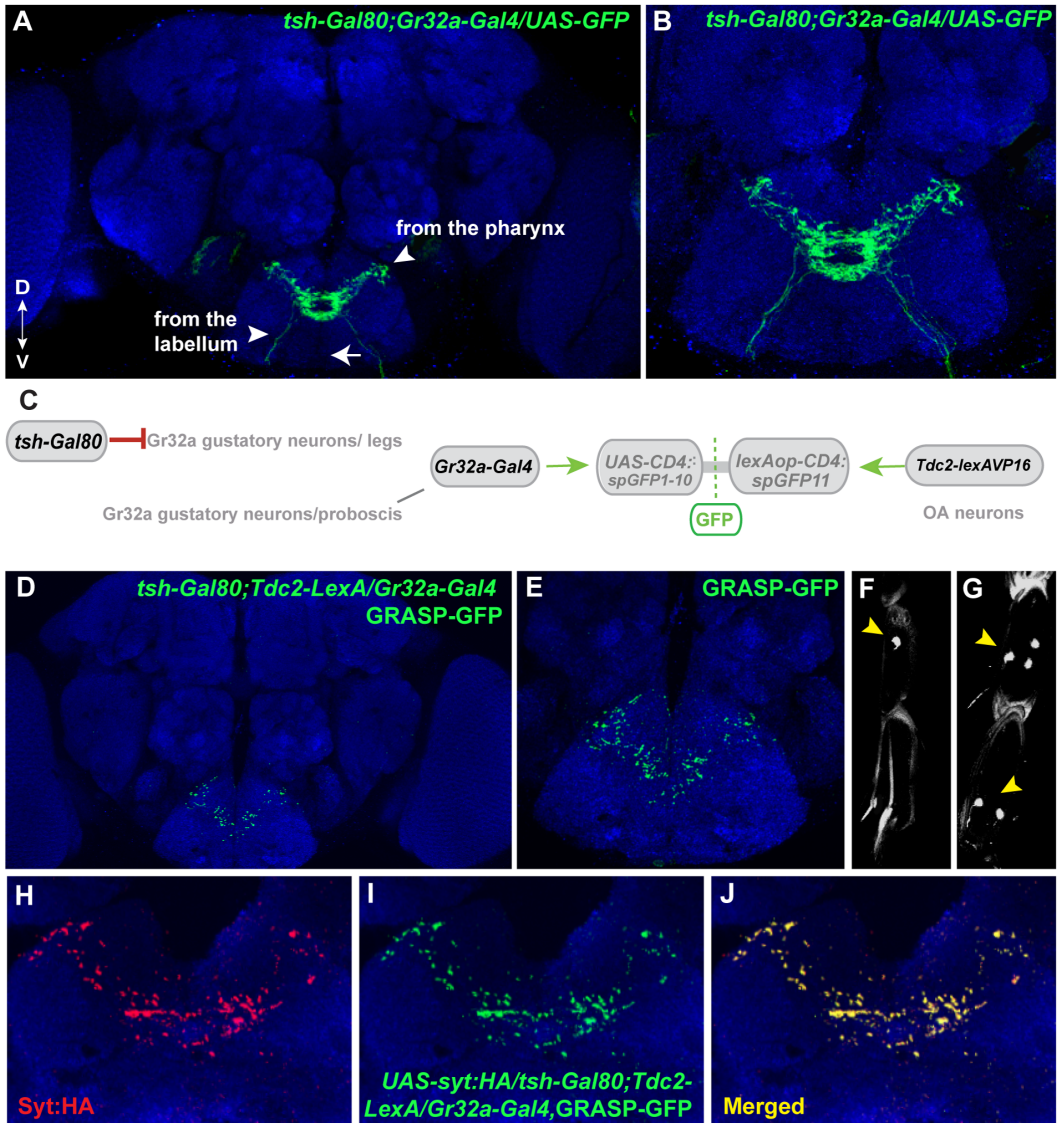
Mouth-specific Gr32a neurons contact OA neurons in the suboesophageal ganglion. (**A**) Axons of Gr32a-expressing neurons located in the mouth identified by immunofluorescence to CD8:GFP in *tsh-Gal80;UAS-CD8:GFP/Gr32a-Gal4* progeny (green, anti-CD8, Invitrogen). Note the absence of axonal projections from the legs via the thoracic ganglion (arrow, compare to Figure 1A). (**B**) Higher magnification of Gr32a mouth neurons expressing CD8:GFP. (**C**) Schematic representation of the GRASP reporter lines combined with the *tsh-Gal80*;*Gr32a-Gal4* and *Tdc2-lexA* driver lines. Gal80 driven by the *tsh-Gal80* line prevents Gal4 activity and subsequent expression of the *UAS-CD4::spGFP1-10* GRASP reporter. (**D–E**) Two different confocal image magnifications of a male brain with the same number of optical sections as in panel A. A reduced amount of GRASP-mediated GFP reconstitution is observed reflecting Gr32a neurons located only in the mouth expressing CD4::spGFP1-10 and OA neurons expressing CD4::spGFP11. GRASP reconstitution is detected by immunofluorescence using rabbit monoclonal GFP antibody (green; Life Technologies). (**F–G**) *tsh-Gal80* blocks GFP expression in Gr32a-expressing leg neurons. Less than one neuron per leg of *UAS-nlsGFP; teashirt-Gal80/Gr32a-Gal4* progeny is observed (arrowhead, 0.38 neurons per front leg, n = 8), versus males without Gal80 expression (arrowhead, 5 neurons per front leg, n = 8). (**H–J**) Optical sections of a female brain (*UAS-syt:HA; tsh-Gal80;UAS-CD8:GFP/Gr32a-Gal4*) at higher magnification showing GRASP-mediated GFP reconstituted expression (I), syt:HA localization (H) and clear overlap or close association at synaptic-like puncta in the merged channel (J).

To identify subpopulation-specific synaptic contacts between Gr32a and OA neurons, we used the *teashirt-Gal80* line in combination with the GRASP system. Recent studies using the Gr32a-Gal4 driver to express GFP indicated at least 38 neurons in the mouth (19 neurons per labial palp) and 11 neurons located in the legs express the reporter [36], [38]. Adding the *teashirt-Gal80* transgene significantly blocked Gal4-mediated activation in the thoracic region resulting in a reduction of GFP expression in the SOG. Thoracic ganglia neuronal projections via the cervical connective are reduced or absent (arrowhead in Fig. 1A, compare Figure 1A to Figure 6A). The reduction of GFP-expression in leg sensory neurons of *UAS-nlsGFP; tsh-Gal80/Gr32a-Gal4* progeny (0.38 neurons per front leg, n = 8), versus males without Gal80 expression (5 neurons per front leg, n = 8) is shown in Figure 6F,G.

With the addition of *teashirt-Gal80* to restrict split-GFP expression to mouth Gr32a neurons, GFP reconstitution is visible in a highly reproducible pattern that appears to be part of the GRASP reconstituted pattern observed when the entire Gr32a-Gal4 expressing population is labeled (compare 6D with 1E). Furthermore, GFP reconstitution co-localizes with the *UAS-syt:HA* reporter added to visualize the presynaptic terminals of Gr32a-expressing neurons. (Figure 6H–J). As Gr32a and OA neuronal function strongly influence male-selective social behaviors, the GRASP patterns of male and female progeny were carefully examined. No apparent sex-specific differences were observed. Results from these experiments suggest that distinct behavioral responses to sex pheromone(s) are provided by separate subsets of Gr32a-expressing chemosensory neurons, in both cases involving potential direct reinforcement by OA.

### Cell-specific refinement of octopamine neuron connections to Gr32a neurons

**Figure 7:**
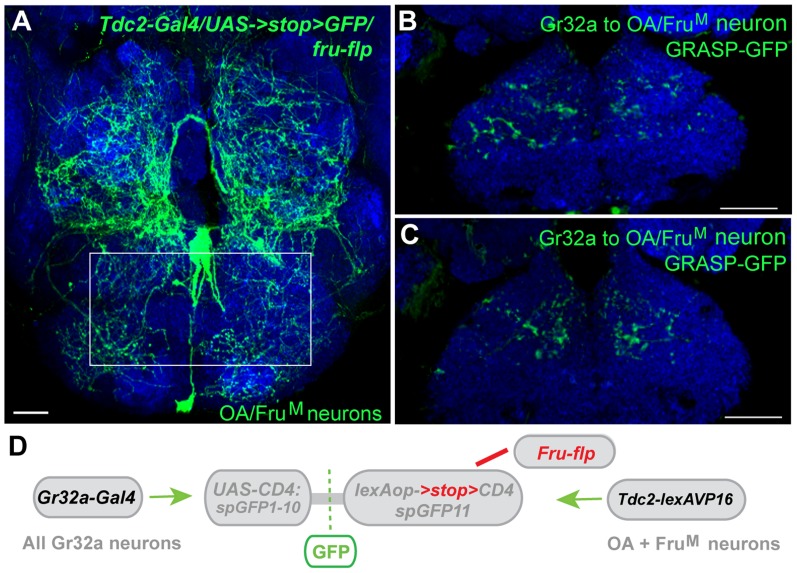
Gr32a neurons anatomically contact three Fru^M^-OA neurons. (**A**) The morphology of three Fru^M^-OA neurons located in the suboesophageal ganglion identified by immunofluorescence to CD8:GFP in *Tdc2-Gal4/UAS->stop>CD8:GFP;fru^FLP^* progeny (green, anti-GFP, Life Technologies). The box outlines the area of putative synaptic connections observed in B and C. Scale bar represents 20 µM. (**B, C**) Two different optical sections of a male brain exhibiting GRASP-mediated GFP reconstitution as a result of Fru^M^-OA neurons expressing CD4::spGFP11 and the entire Gr32a neuron population expressing CD4::spGFP1-10. GRASP reconstitution is detected by immunofluorescence using rabbit monoclonal GFP antibody (green; Life Technologies). Scale bar represents 30 µM. (**D**) Schematic representation outlining the GRASP reporter lines combined with *fru^FLP^*, *Gr32aVP16-Gal4*, and *Tdc2-lexA*. The FLP recombinase enzyme driven by *fru^FLP^* excises the stop codon and permits expression of the *lexAop2>stop>::spGFP11* GRASP reporter. Scale bar represents 30 µM.

We previously demonstrated that three OA neurons express the male form of Fruitless (Fru^M^), a neural sex determination factor that is a key determinant of male patterns of courtship and aggression (Figure 7A) [21], [42], [43]. The necessity of Fru^M^ expression in this small subset of OA neurons was evident as the absence of Fru^M^ resulted in an increase in male-male courtship in an aggression setting [21]. These results suggested that sexual specification of certain OA neurons might be involved in reliably establishing mate selection (or reliably suppressing conspecific male-male courtship). To determine if Gr32a-expressing neurons establish synaptic contacts with Fru^M^-OA neurons, *Tdc2-LexA* was used in conjunction with the recently generated restrictable split-GFP component, *lexAop2->stop>CD4::spGFP11* (María Paz Fernández, unpublished data). Selectively activating split-GFP11 expression in Fru^M^ neurons was achieved through the production of the FLP enzyme in Fruitless-expressing neurons via the *fru^FLP^* [44] line and putative synaptic connections were observed in male and female brains also expressing *Gr32a-Gal4* driven *UAS-CD4::spGFP1-10* (Figure 7B–D). At this time, we cannot simultaneously restrict Gr32a-expressing and OA neuronal populations or as yet quantify any sex-specific connection differences that may exist. However, our experiments indicate the Fru^M^-OA neurons that account for increases in male-male courtship are anatomically connected to Gr32a neurons and these may form a microcircuit that contributes to the context-specificity of male courtship behavior.

## Discussion

Studies on animal behavior have been ongoing for decades and these have resulted in identifying pheromones, hormones and neurohormones, neurons, circuits and more recently, genes, that cause or contribute to the expression of social behavior. Yet a broad gap still exists between the identification of neurons and circuits suspected of involvement in specific behaviors and an understanding of how these circuits orchestrate the many context-dependent complex decisions animals routinely make in their daily lives. In this study, we demonstrate a direct early sensory link to a neuromodulatory-signaling element concerned with male aggression and courtship behavior and show that the two are interconnected in the suboesophageal ganglion. Our results show that sensory neurons expressing Gr32a, a widely distributed gustatory receptor that plays a critical role in male social behaviors [14]–[17], relays primary sensory information to the SOG where octopaminergic interneurons are contacted. The high density of putative GRASP connections we observe between receptor neurons expressing Gr32a, 22e, and 59b, and OA neurons in the SOG (these are co-expressed in a subset of the labellar sensory receptor neuron pool) [36]), suggests that amine-dependent modulatory steps may serve as important second order components in connecting signals from taste receptor neuron subtypes to taste-evoked behavior in flies [31], [45] (in vertebrates and other invertebrate systems see [46], [47], [48]). A separate study also identified putative synaptic connections between Gr32a axons and the total population of Fru^M^-expressing neurons [17]. Whether Gr32a-expressing neurons solely contact the OA-Fru^M^ neurons or whether they contact additional Fru^M^ neurons remains to be determined. We do observe regions of Gr32a-driven syt:HA expression without GFP reconstitution to OA neurons suggesting the Gr32a-expressing neuron population likely contacts additional neuron subsets.

The Gr32a receptor is categorized as a contact-based chemoreceptor and is required for physiological responses to caffeine and other aversive, bitter-tasting compounds [36], [49]–[51]. Gr32a is also reported to mediate the behavioral effects of the male pheromone *(z)*-7-tricosene and regulate interspecies courtship [16], [17]. *(z)*-7-tricosene application to male legs evoked an increase in Ca^2+^ signaling in OA neurons (Andrews and Certel, unpublished data), although we were unable to identify a reliable response to *(z)*-7-tricosene in Gr32a foreleg neurons at this time. Reconciling behavioral and physiological roles of Gr32a-expressing leg and labellar neurons to individual CHCs will require further investigation. Nevertheless, application of male CHCs to male legs evokes significant increases in Ca^2+^ signaling in OA neurons and this response is eliminated in males with ablated Gr32a neurons (Figure 4). These results support the behavioral data that indicates male aggression is promoted through the *Gr32a* receptor (this study and [16] and suggests that at least a portion of the sensory information mediated by Gr32a receptor-bearing sensory neurons and OA modulatory interneurons operate in a single circuit.

The manipulation of neuronal populations by altering the expression of single molecular products like the Gr32a gustatory receptor or one of the monoamines, commonly yields multiple behavioral phenotypes [14]–[16] indicating that such populations are heterogeneous in function. Separation of the grouped neurons into small subgroups can clarify the roles of these neurons in behavior and ultimately is essential in defining the circuitry involved. Recent findings indicate the tarsal Gr32a neurons are necessary to mediate species recognition [17]. Our data demonstrate that the foreleg tarsi and mouth populations of Gr32-expressing neurons may exert separable functional differences on male aggression and courtship behavior with both populations involving direct reinforcement by OA. Although Gr32a-expressing neurons do not exhibit any obvious sexual dimorphism, it has been postulated that their postsynaptic targets are sexually dimorphic [14]. With the increasing genetic capabilities of individual neuron manipulation, it will be interesting to determine if sexually dimorphic connectivity between single Gr32a and Fru^M^-OA neurons regulate distinct differences in social behaviors. Results from further anatomical studies could provide insight into how potential sexual modification of OA signaling links chemosensory input to sex-specific behavioral output.

Neural networks mediating ever-changing environmental stimuli, context-specific social behavior, and internal states challenge us with the overwhelming structural and functional complexity of their interactions. To attempt to reduce network complexity, one common approach is to define network subunits and demonstrate their functional role by selective removal. It is well known that amine neurons can signal through hormonal volume transmission and act on targets at a distance [52], [53]. However, biogenic amines are also released synaptically and act on local targets [54]–[58]. Whether amine neurons function in separate modulatory circuits that run parallel to and interact with hard-wired circuitries directing behavior, or whether they are an integral part of such circuitry remains to be determined. However, understanding the presynaptic sources or postsynaptic targets of OA neurons should provide useful insight into the “structural” embeddedness of single cells within a network. An anatomical analysis of individual components will be necessary as proximity-based neuron groupings break down with the addition of cell-specific markers (like Fru^M^) and within amine neuron populations [59]. Network anatomical characterization that includes neuromodulatory neurons may also provide insight into the reinforcing or opposing actions of amines through second amines or peptide modulators [60], [61]. For example, Burke et al., recently demonstrated plausible sites of synaptic contact between OA and DA neurons in the *Drosophila* mushroom body and a role for OA in providing appetitive reinforcement by OA receptor-mediated actions on DA neuron populations [29]. Our study offers a valuable framework in which to undertake the characterization of sensory-driven neural circuits and the underlying neuromodulation of sexually dimorphic patterns of social behavior.

## Materials and Methods

The following strains were used in this study: *Gr32a* ^−/−^ [15], *Gr32a-Gal4* [62], *Gr32a-I-GFP* [33], *UAS-DTI* (obtained from Leslie Stevens), *UAS-transformer* (BL 4590), *UAS-synaptotagmin:hemagglutinin* (*UAS-syt:HA* [34]), *w* ^+^ *tβh^nM18^* [23], *w* ^+^ *tβh^M6^* [23], *dTdc2-Gal4* [28], *tsh-Gal80* (provided by Julie Simpson) [63], *UAS-mCD8:dsRed* (obtained from Liz Gavis), *lexAop-CD4::spGFP11* and *UAS-CD4::spGFP1-10* [27], *UAS-Red Stinger* (BL 8545), *UAS-hid UAS-Red Stinger*, *UAS-Denmark* (BL 33063) [64], *fru^FLP^* [44], *20XUAS-6XGFP-Myc* (a gift from Steve Stowers, BL 52262), *UAS-GCaMP3.0* (BL-53742), *20XlexAop2-IVS-GCaMP6s* (BL 44274) and the Canton-S strain from the Bloomington Stock Center, Bloomington, IN.

### Generation of transgenic lines

The *dTdc2-lexA:VP16* transgenic line was generated by cloning the same regulatory region as described previously [28] into the pBS_LexA::VP16_SV40 vector. In the previous construct, the GAL4 was inserted immediately before the coding start, and the entire construct (genomic segments interrupted by Gal4) was inserted into the polylinker of pCaSpeR4 [28]. To generate the *dTdc2-lexA:VP16* construct, genomic DNA containing the region −3459 to +4530 was amplified with the Expand Long Template PCR system (Roche Applied Science). Fragment “A” of the *dTdc2* genomic region was amplified using the following primers, Tdc2A- Forward: GTCGCGGCCGCAAAAGTTATTGCACATTG, Tdc2A-Reverse: GGCCGGCCGTTTCGGTAGGTTTTCCAAATC, and fragment “B” with the following primers, Tdc2B Forward: GTCGGGCCCATGGACAGCACCGAATTTC, Tdc2B-Reverse: GGCCGCGGCCGCTTAGAACATATCGAGTTG. The *dTdc2* fragment A PCR product was inserted directly into the pBS-LexA::VP16_SV40 vector via the Eag1 site. Fragment B was first inserted into the TOPO vector and digested with Apa1, followed by ligation into to pBS-Tdc2fragmentA-LexA::VP16_SV40 using the Apa1 site on the vector. The fragment containing Tdc2 fragment A+ the *LexA* coding region+*dTdc2* fragment B was subcloned into the Not1 site of pCaSpeR4.

The *lexAop2-FRT-STOP-FRT-::spGFP11* line was generated by amplifying the spGF11 fragment through PCR from the previously described pLOT plasmid [27]. The FRT-STOP-FRT cassette was amplified from the pJFRC177 plasmid (#32149, AddGene) and both the STOP cassette and the spGFP11 fragment were cloned downstream of the 13XLexAop2 sequence in pJFRC19 (#26224, AddGene). The amplified fragments were verified by sequencing. Transgenic flies were raised by standard procedures and lines screened for appropriate expression.

### Immunohistochemistry

Adult male and female dissected brains were fixed in 4% paraformaldehyde (Electron Microscopy Sciences) for 25 minutes and labeled using a modification of protocols previously described [23]. The following primary antibodies were used: rabbit anti-GFP monoclonal (1∶200) (Life Technologies, G10362), mouse anti-GFP (1∶200) (Invitrogen, A-11120, Lot 764809), rabbit anti-FruM (1∶2000) [43], rat anti-CD8 (1∶100), rat anti-HA (Roche, 1∶1000), mAb nc82(anti-bruchpilot) (1∶30) [65], anti-Tβh (1∶400) [66]. Secondary antibodies include Alexa Fluor 488-conjugated goat anti-rabbit, Alexa Fluor 488-conjugated donkey anti-mouse, Alexa Fluor 594-conjugated donkey anti-mouse, Alexa Fluor 594-conjugated goat anti-rabbit, Alexa Fluor 647-conjugated donkey anti-mouse (Invitrogen). Cross-adsorbed goat anti-rabbit fluorescein-conjugated secondary antibodies were used in multi-labeling experiments. Images were collected on an Olympus Fluoview FV1000 laser scanning confocal mounted on an inverted IX81 microscope and processed using ImageJ (NIH) and Adobe Photoshop (Adobe, CA).

### Behavioral assays

All fly strains were reared on standard fly food (medium containing agar, glucose, sucrose, yeast, cornmeal, propionic acid, and Tegosept). Flies were grown in temperature- and humidity-controlled incubators (25°C, 50% humidity) on a 12-h light/dark cycle. To collect socially naïve adults, pupae were isolated in individual 16×100-mm glass vials containing 1.5 ml of food medium. Upon eclosion, flies were anesthetized with CO_2_, painted on the thorax with acrylic paint for identification and returned to their isolation vials to allow for recovery from anesthesia a full 24 hours before testing.

### Calcium imaging

Live brain preparations were made by anesthetizing a fly on ice followed by placement within a pipette tip with the head protruding. The pipette was then sealed with nail polish and allowed to dry. Flies thusly secured were placed in a 1 mL well for electrophysiology at an angle and the region containing the head was flooded with 400 µL of oxygenated HL3 solution. Removal of the proboscis and front of the head cuticle allowed for imaging. Each preparation was equilibrated for 5 min after proboscis and cuticle dissection. Male cuticular hydrocarbon extract (hexane extract from 150 male flies 3 days post eclosure), (*z*)-7-tricosene (Cayman Chemical #9000313 Lot# 0406404-32), or quinine (Sigma-Aldrich #6119-47-7 Lot # STBD3004V) dissolved in oxygenated HL3 solution was administered via syringe into the rear of the pipette tip. Administration of each compound occurred a minimum of 15 seconds apart. Flies received either male cuticular extract or (*z*)-7-tricosene first, followed by quinine. Analysis of ΔF/F values in regions of interest was calculated using Fiji and Prism 6.0.

### Image analysis

Epifluorescene images were acquired at the rate of 1 image/.750s by Hamamatsu camera (ORCA ER series, model C4742-95-12ERG). Acquired images were registered (StackReg plugin, Fiji software) and regions of interest were selected within the suboesophageal ganglion. Image processing and analysis was accomplished with ImageJ version 1.44/Fiji version 1.43. Image subtraction was performed in Fiji using the image calculator. Intensity tables were exported to excel and (ΔF−F)/F calculated for each series of images. Traces were generated in Prism 6.0. Peak analysis was performed between regions no more than 5 seconds post compound administration (for post CHC) and no later than 4 seconds prior to compound administration (for pre-CHC).

### Aggression and courtship paradigms

Aggression assays were performed in individual chambers of 12-well polystyrene plates containing a food cup in the center [67]. 4–5 day old males were transferred in pairs to assay chambers by aspiration. Experiments were performed at 25°C in a humidity controlled room (50%). Fights were videotaped for 90 minutes and lunges counted for 30 minutes from the first lunge unless otherwise specified. The time between introduction into the chamber and the onset of aggression (first lunge) was defined as the fighting latency. Lunging behavior was determined as previously described [68]. Courtship assays were performed in a 12 well polystyrene plate (VWR #82050-930) with one Canton S virgin female (aged 7–10 days) and one 4–5 day old male. The period between introduction into the courtship chamber and the first male wing extension (singing) was defined as courtship latency.

## Supporting Information

Figure S1 Characterization of the *Tdc2-LexA* line. (**A**) GFP expression drive by *Tdc2-LexA* in the adult brain maintains the same pattern as the *Tdc2-Gal4* driver. The SOG region shown in panels B and C from a separate brain is outlined with the white box. (**B–C**) Complete overlap is observed between Tβh immunoreactivity and GFP in Tdc2-lexA SOG neurons (*Tdc2-lexA;lexA-rCD2:GFP* progeny). (**D**) GFP expression driven by the Tdc2-LexA line in a cluster of SOG neurons visualized in *Tdc2-LexA;lexAop-rCD4:GFP* progeny. Extensive arborizations (arrows) of Tdc2 neurons within the SOG are visualized in a series of optical sections ventral to the cell bodies (*Tdc2-LexA;lexAop-rCD4:GFP* progeny).
(PDF)

Figure S2 Single GRASP component control brains demonstrate an absence of GFP expression. (**A–D**) Control brains were imaged for immunofluorescence against GFP in brains containing one component of the GRASP system. (**A**) No signal was observed in *Gr32a-Gal4/UAS-CD4::spGFP1-10* controls. (**B**) Fluorescence was not detected in *Tdc2-lexA:VP16/lexAop2-CD4::*spGFP11 control brains. (**C**) The *UAS-CD4::spGFP1-10* GRASP component driven by *Gr32a-Gal4VP16* did not generate a signal. (**D**) The addition of an flp-out stop codon in progeny containing *Tdc2-lexA:VP16/lexAop2->stop>CD4::*spGFP11 did not result in detectable fluorescence. All brains were labeled with rabbit monoclonal GFP, Life Technologies. Scale bar represents 20 µM.
(PDF)

Figure S3 Gr22e and Gr59b neurons contact OA neurons in the suboesophageal ganglion. (**A**) GRASP-mediated GFP reconstitution specifically in the SOG is observed between Gr22e neurons expressing CD4::spGFP1-10 and synaptotagmin:hemagglutinin (*UAS-syt:HA*) (red, anti-HA) and OA neurons expressing CD4::spGFP11. GRASP reconstitution is detected by immunofluorescence using a monoclonal GFP antibody (green, Invitrogen, A-11120, Lot 764809). (**B–D**) Optical sections at higher magnification showing GRASP-mediated GFP reconstituted expression (D), syt:HA localization (C) and clear overlap or close association at synaptic-like puncta in the merged channel (B). (**E**) GRASP-mediated GFP reconstitution between Gr59b neurons expressing CD4::spGFP1-10 and *UAS-syt:HA* (red, anti-HA) and OA neurons expressing CD4::spGFP11. Regions in the SOG with only syt:HA expression are indicated (arrow) in addition to GFP-reconstitution contacts that show co-localization with syt-HA expression. (**F–H**) Higher magnification view of optical sections with GRASP-mediated GFP reconstitution (J), syt:HA localization (I), and the observed overlap in punctate patterns (H). Scale bars represent 20 µM.
(PDF)

Figure S4 GRASP-reconstitution between Gr47a neurons and OA-expressing neurons is not observed. (**A**) The *Gr47a-Gal4* line drives GFP expression via the *UAS-CD8:GFP* reporter in the SOG (arrow). (**B**) The single GRASP line *UAS-CD4::spGFP1-10* is expressed by *Gr47a-Gal4* and detected by a polyclonal rabbit anti-GFP that recognizes this split-GFP fragment (Invitrogen, A6455). (**C**) GRASP-mediated GFP reconstitution was not observed between Gr47a neurons expressing CD4::spGFP1-10 and OA neurons expressing CD4::spGFP11 (monoclonal GFP, Invitrogen, A11120, Lot 764809).
(PDF)

Figure S5 Tdc2-expressing neurons are ablated by *UAS-hid UAS-DsRed* expression. (**A**) Expression of the rate-limiting enzyme, Tyrosine β-hydroxylase, is detected in OA-expressing SOG neurons in *Tdc2-Gal4/+* control brains (anti-Tβh, [66]). (**B**) Octopamine neurons are eliminated in *Tdc2-Gal4/UAS-hid UAS-DsRed* progeny as assayed by the absence of DsRed and Tyrosine β-hydroxylase production. Scale bar represents 20 µM.
(TIF)

Figure S6 Eliminating OA production alters a subset of Gr32a axonal projections. (**A**) GFP expression in a heterozygous control adult brain *(tβh^nM18^/+;Tdc2-Gal4;20XUAS-6XGFP-Myc)*. The Gr32a-expressing neurons located in the tarsi, labellum, and mouthparts terminate in the SOG. (**B**) Schematic representation of the adult brain with Gr32a-expressing axonal arborizations. (**C–D**) Confocal sections of OA deficient male brains (*(tβh^nM18^;Tdc2-Gal4;20XUAS-6XGFP-Myc)*. When OA production is eliminated throughout development, a subset of Gr32a axon projections terminate in the antennal lobe region (arrow). (**E**) Schematic representation of the adult OA deficient brain with a subset of Gr32a-expressing axons terminating in the antennal lobe region. Scale bar represents 30 µM.
(TIF)

Figure S7 Defects in aggressive behavior parameters in Gr32a-expressing and OA deficient males. (**A–C**) Experimental males without Gr32a-expressing neurons *(UAS-DTI;Gr32a-Gal4)* do not exhibit aggressive behavior when paired with control males. (**A**) Males without Gr32a-expressing neurons display significantly fewer lunges than control males *(+/Gr32a-Gal4)*. (**B**) Control males initiated aggression as measured by the first lunge in all assays, n = 15. (**C**) The latency to first lunge by control males is similar in pairings with experimental and control males (Figure 3). (**D**) The number of lunges by experimental *tβh^nM18^;UAS-DTI/+*; *Gr32a-Gal4/+* males was significantly less than exhibited by control males (*tβh^M6^*) or males without OA (*tβh^nM18^*) (****p<0.0001, **p = 0.003). (**E–G**) Aggressive behavior or the component patterns that make up aggressive behavior are commonly quantified for a given period of time from the moment that pairs of flies are placed into a fight chamber (**E**, upper panel). This method of scoring does not take into account any substantial differences in the latency to begin fighting. Given the observed latency to initiate the fights, we quantified the number of lunges performed by each pair of males during a 30-minute period starting from the onset of aggression (lower panel). (**F**) If fights without lunges are scored as “zeros”, the numbers of lunges seen in fights between pairs of *tβh^nM18^* males are significantly lower than the numbers seen in the genetic controls. One outlier value of 416 is observed in a *tβh^nM18^* pairing. In this comparison with fights that do not exhibit fighting, *tβh^M6^* and *tβh^nM18^* are statistically different with the inclusion or absence of the outlying value (Mann-Whitney test, p value with outlier = 0.0049, p value without outlier = 0.0023) (**G**) When pairs that did not display lunges are excluded in the quantification, significant differences are not found in the lunge frequency between *tβh^nM18^* and *tβh^M6^* male pairs. One outlier value of 416 is observed in a *tβh^nM18^* pairing. In this panel *tβh^M6^* and *tβh^nM18^*are not statistically different with the inclusion or absence of the outlying value (Mann-Whitney test, p value with outlier = 0.2193. p value without outlier = 0.1327. Both >0.5). (**H**) Elevated male-male courtship occurs when lunge number is low in male pairs without OA (*tβh^nM18^*). The three columns, <10, 10–25, and >25 represent the observed number of lunges per fight. For each assay, the number of wing extensions/singing was divided by the number of lunges resulting in the average ratios per column.
(PDF)

Figure S8 *tsh-Gal80* blocks Gal4-mediated expression in the leg. (**A**) Gr32a neurons expressing GFP in the labellum of *tsh-Gal80;Gr32a-Gal4/UAS-CD8:GFP* progeny (arrow). (**B**) The addition of *UAS-DTI* ablates the Gr32a-expressing labellar neurons. (**C**) Gr32a leg neurons still maintain GFP expression in *UAS-DTI/tsh-Gal80;Gr32a-Gal4/UAS-CD8:GFP* progeny. Scale bar represents 20 µM.
(TIF)

Table S1 Analysis of male-female courtship in males with ablated mouth Gr32a-expressing neurons. Single male to virgin female courtship parameters measured in control males and males with mouth Gr32a-expressing neurons ablated. Latency to courtship initiation is the time when a singing/wing extension event to the female is first observed after introduction into the courtship chamber. Courtship initiation differences between *UAS-DTI/+* controls, *tsh-Gal80;Gr32a-Gal4* controls, and *UAS-DTI/tsh-Gal80;Gr32a-Gal4* males were significant (Kruskal-Wallis with Dunn's multiple comparison test, **p<0.01, ***p<0.001). However, the delay did not significantly change copulations rates. Due to the extended latency period exhibited by *UAS-DTI/tsh-Gal80;Gr32a-Gal4* males, the copulation rate equals the percentage of males mating in 60 minutes.
(TIF)
