# Supplementary figures and images for: Octopamine Neuromodulation Regulates Gr32a-Linked Aggression and Courtship Pathways in Drosophila Males
Source: PLoS Genet. 2014 May 22;10(5):e1004356. doi: 10.1371/journal.pgen.1004356 (PMC4031044; doi:10.1371/journal.pgen.1004356)

Supplementary Figure S1 (Andrews et al.,). Characterization of the *Tdc2-LexA* line

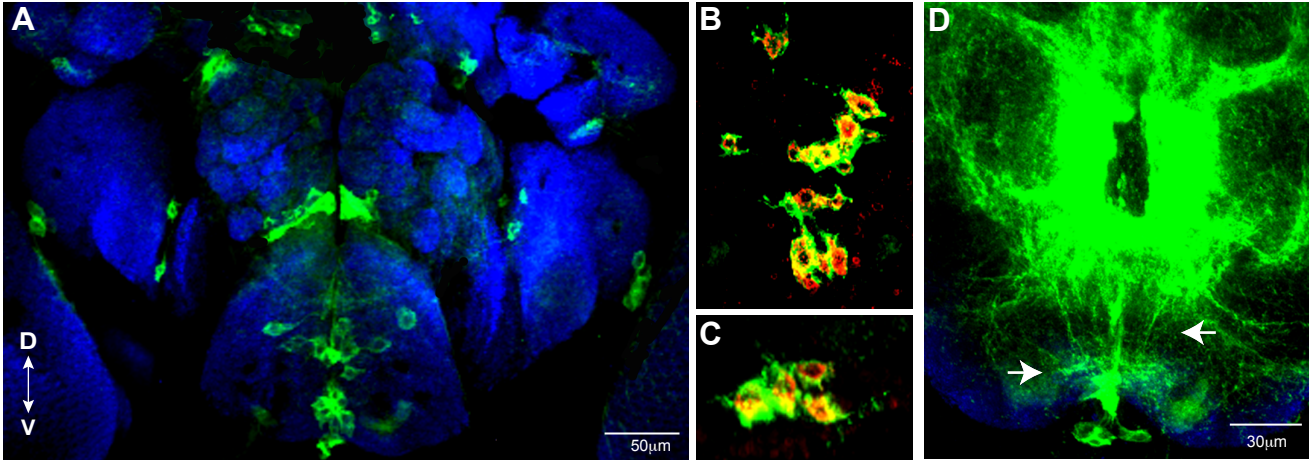

Supplement: Figure S1 — Characterization of the Tdc2-LexA line. (A) GFP expression drive by Tdc2-LexA in the adult brain maintains the same pattern as the Tdc2-Gal4 driver. The SOG region shown in panels B and C from a separate brain is outlined with the white box. (B–C) Complete overlap is observed between Tβh immunoreactivity and GFP in Tdc2-lexA SOG neurons (Tdc2-lexA;lexA-rCD2:GFP progeny). (D) GFP expression driven by the Tdc2-LexA line in a cluster of SOG neurons visualized in Tdc2-LexA;lexAop-rCD4:GFP progeny. Extensive arborizations (arrows) of Tdc2 neurons within the SOG are visualized in a series of optical sections ventral to the cell bodies (Tdc2-LexA;lexAop-rCD4:GFP progeny). (PDF) [file pgen.1004356.s001.pdf]

Andrews et al., Figure S4. Gr47a-expressing neurons do not contact OA neurons

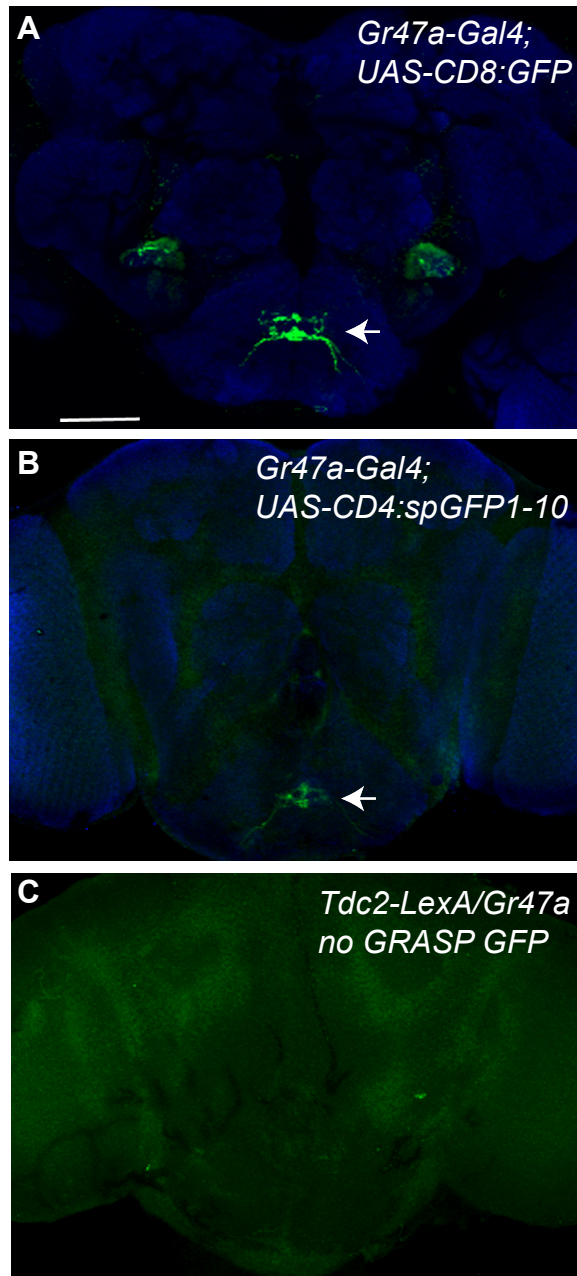

Supplement: Figure S4 — GRASP-reconstitution between Gr47a neurons and OA-expressing neurons is not observed. (A) The Gr47a-Gal4 line drives GFP expression via the UAS-CD8:GFP reporter in the SOG (arrow). (B) The single GRASP line UAS-CD4::spGFP1-10 is expressed by Gr47a-Gal4 and detected by a polyclonal rabbit anti-GFP that recognizes this split-GFP fragment (Invitrogen, A6455). (C) GRASP-mediated GFP reconstitution was not observed between Gr47a neurons expressing CD4::spGFP1-10 and OA neurons expressing CD4::spGFP11 (monoclonal GFP, Invitrogen, A11120, Lot 764809). (PDF) [file pgen.1004356.s004.pdf]

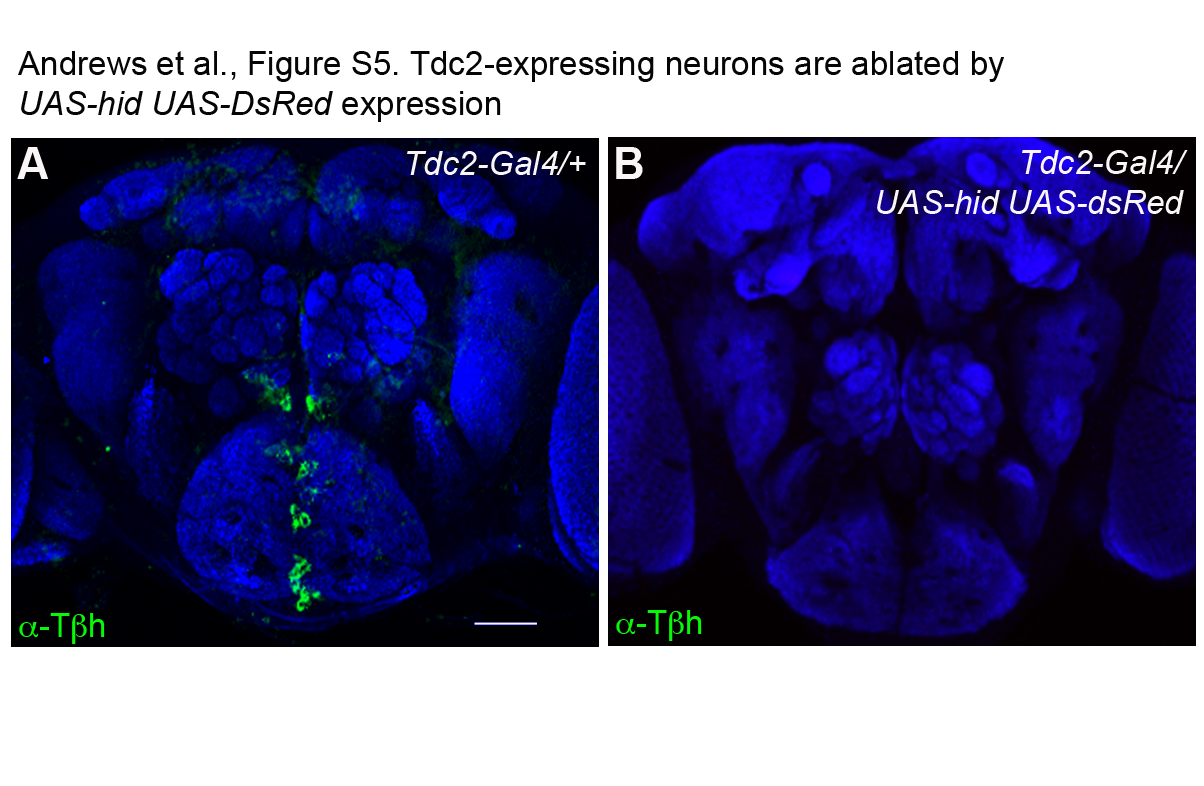

Supplement: Figure S5 — Tdc2-expressing neurons are ablated by UAS-hid UAS-DsRed expression. (A) Expression of the rate-limiting enzyme, Tyrosine β-hydroxylase, is detected in OA-expressing SOG neurons in Tdc2-Gal4/+ control brains (anti-Tβh, [66]). (B) Octopamine neurons are eliminated in Tdc2-Gal4/UAS-hid UAS-DsRed progeny as assayed by the absence of DsRed and Tyrosine β-hydroxylase production. Scale bar represents 20 µM. (TIF) [file pgen.1004356.s005.tif]

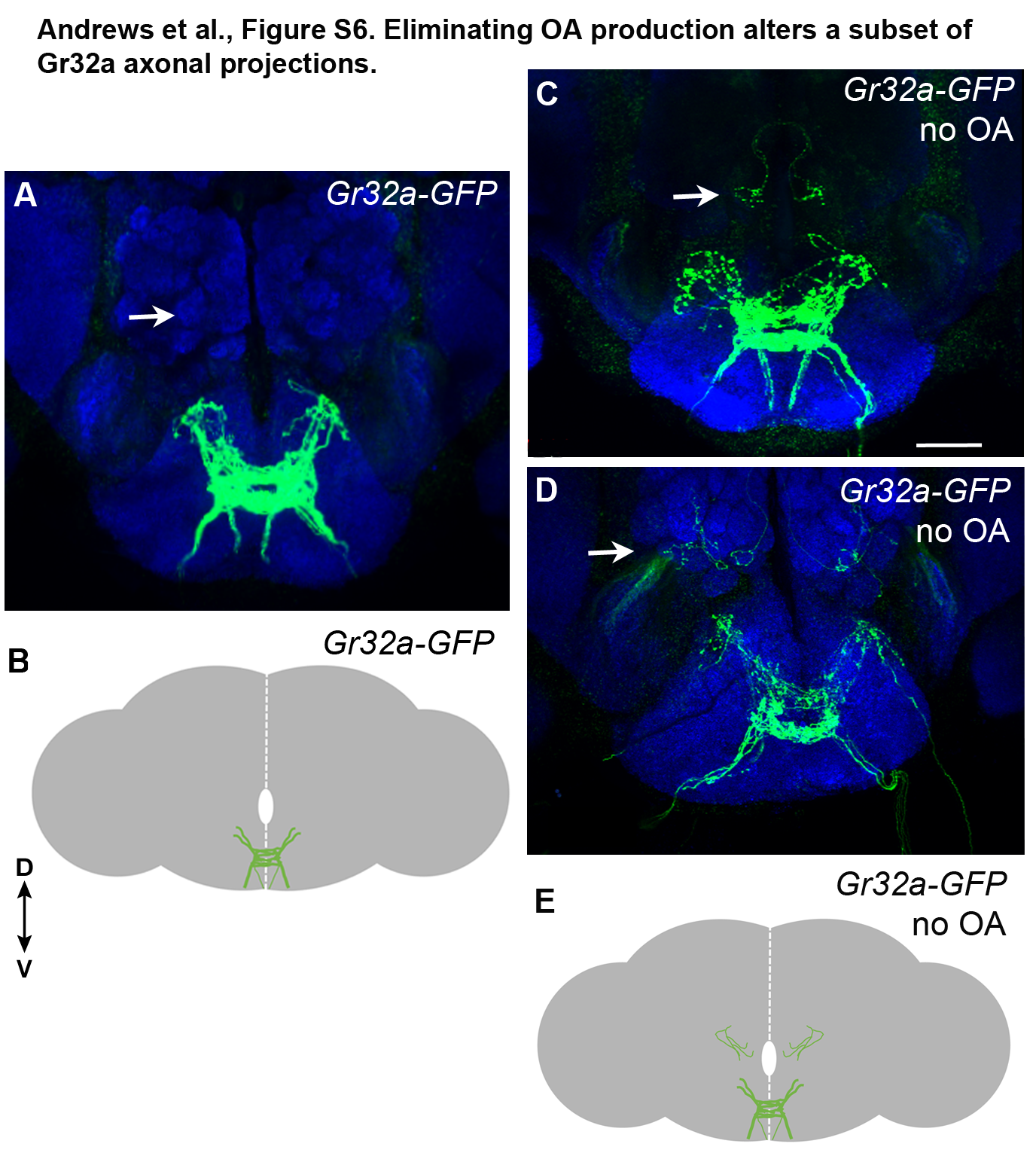

Supplement: Figure S6 — Eliminating OA production alters a subset of Gr32a axonal projections. (A) GFP expression in a heterozygous control adult brain (tβhnM18/+;Tdc2-Gal4;20XUAS-6XGFP-Myc). The Gr32a-expressing neurons located in the tarsi, labellum, and mouthparts terminate in the SOG. (B) Schematic representation of the adult brain with Gr32a-expressing axonal arborizations. (C–D) Confocal sections of OA deficient male brains ((tβhnM18;Tdc2-Gal4;20XUAS-6XGFP-Myc). When OA production is eliminated throughout development, a subset of Gr32a axon projections terminate in the antennal lobe region (arrow). (E) Schematic representation of the adult OA deficient brain with a subset of Gr32a-expressing axons terminating in the antennal lobe region. Scale bar represents 30 µM. (TIF) [file pgen.1004356.s006.tif]

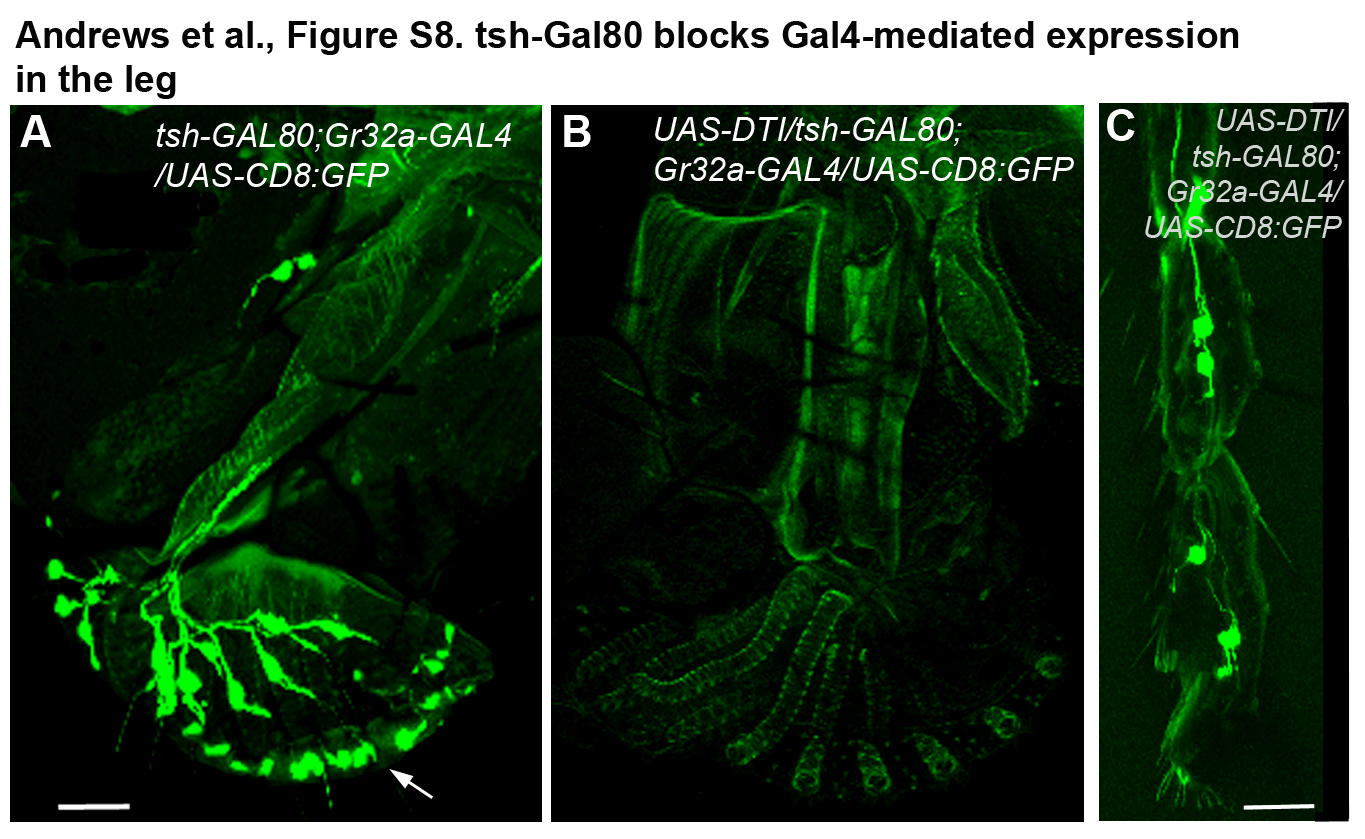

Supplement: Figure S8 — tsh-Gal80 blocks Gal4-mediated expression in the leg. (A) Gr32a neurons expressing GFP in the labellum of tsh-Gal80;Gr32a-Gal4/UAS-CD8:GFP progeny (arrow). (B) The addition of UAS-DTI ablates the Gr32a-expressing labellar neurons. (C) Gr32a leg neurons still maintain GFP expression in UAS-DTI/tsh-Gal80;Gr32a-Gal4/UAS-CD8:GFP progeny. Scale bar represents 20 µM. (TIF) [file pgen.1004356.s008.tif]

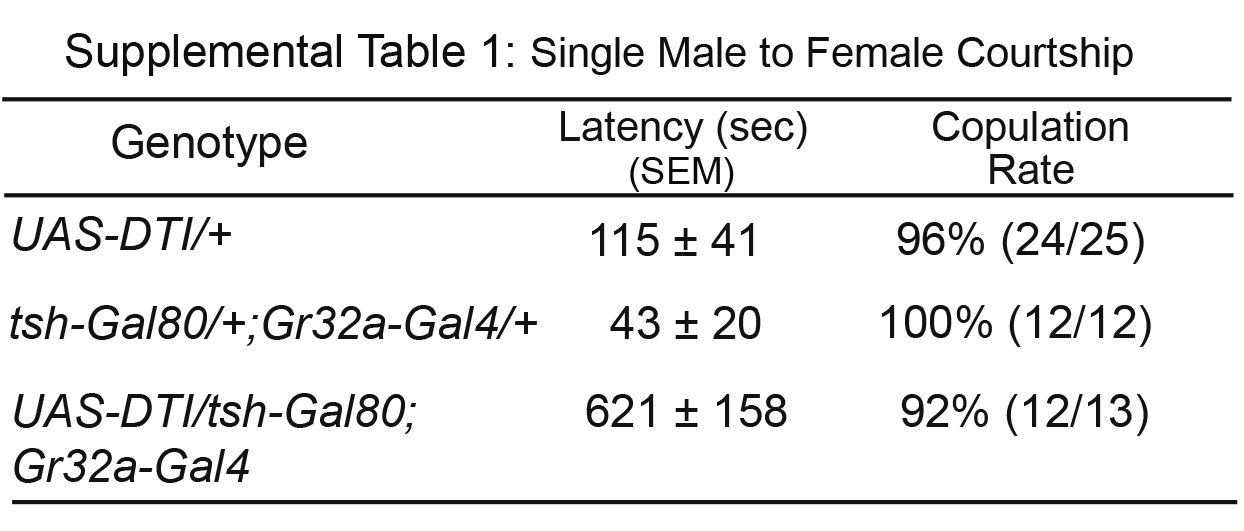

Supplement: Table S1 — Analysis of male-female courtship in males with ablated mouth Gr32a-expressing neurons. Single male to virgin female courtship parameters measured in control males and males with mouth Gr32a-expressing neurons ablated. Latency to courtship initiation is the time when a singing/wing extension event to the female is first observed after introduction into the courtship chamber. Courtship initiation differences between UAS-DTI/+ controls, tsh-Gal80;Gr32a-Gal4 controls, and UAS-DTI/tsh-Gal80;Gr32a-Gal4 males were significant (Kruskal-Wallis with Dunn's multiple comparison test, **p<0.01, ***p<0.001). However, the delay did not significantly change copulations rates. Due to the extended latency period exhibited by UAS-DTI/tsh-Gal80;Gr32a-Gal4 males, the copulation rate equals the percentage of males mating in 60 minutes. (TIF) [file pgen.1004356.s009.tif]
