# Supplementary material for: Octopamine Neuromodulation Regulates Gr32a-Linked Aggression and Courtship Pathways in Drosophila Males
Source: PLoS Genet. 2014 May 22;10(5):e1004356. doi: 10.1371/journal.pgen.1004356 (PMC4031044; doi:10.1371/journal.pgen.1004356)

Supplemental Figure 2 (Andrews et al.,). Single GRASP component control brains demonstrate an absence of GFP expression

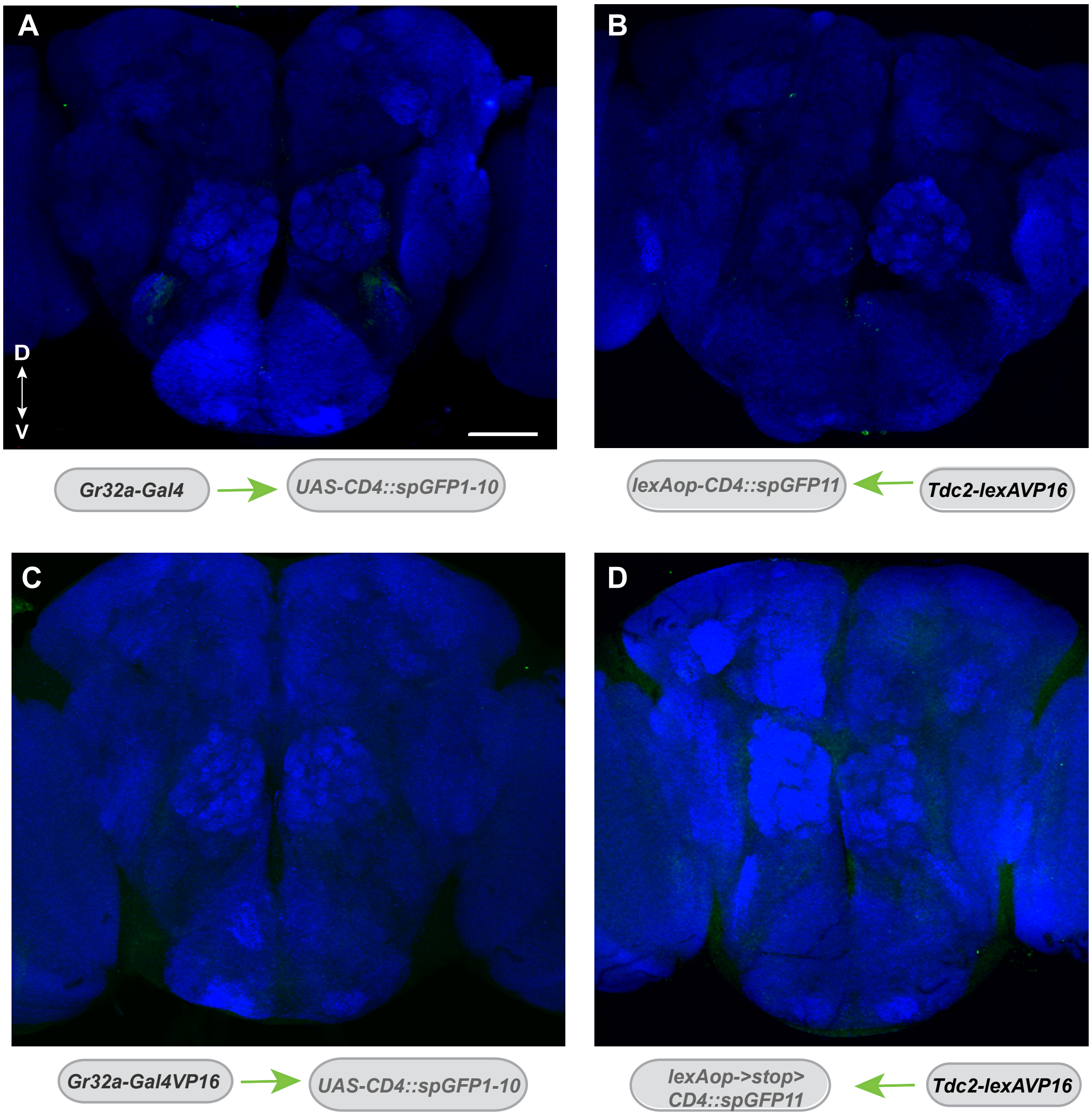

Supplement: Figure S2 — Single GRASP component control brains demonstrate an absence of GFP expression. (A–D) Control brains were imaged for immunofluorescence against GFP in brains containing one component of the GRASP system. (A) No signal was observed in Gr32a-Gal4/UAS-CD4::spGFP1-10 controls. (B) Fluorescence was not detected in Tdc2-lexA:VP16/lexAop2-CD4::spGFP11 control brains. (C) The UAS-CD4::spGFP1-10 GRASP component driven by Gr32a-Gal4VP16 did not generate a signal. (D) The addition of an flp-out stop codon in progeny containing Tdc2-lexA:VP16/lexAop2->stop>CD4::spGFP11 did not result in detectable fluorescence. All brains were labeled with rabbit monoclonal GFP, Life Technologies. Scale bar represents 20 µM. (PDF) [file pgen.1004356.s002.pdf]
