# Supplementary material for: Octopamine Neuromodulation Regulates Gr32a-Linked Aggression and Courtship Pathways in Drosophila Males
Source: PLoS Genet. 2014 May 22;10(5):e1004356. doi: 10.1371/journal.pgen.1004356 (PMC4031044; doi:10.1371/journal.pgen.1004356)

Supplementary Figure S3 (Andrews et al.,). Gr22e and Gr59b neurons contact OA neurons in the suboesophageal ganglion.

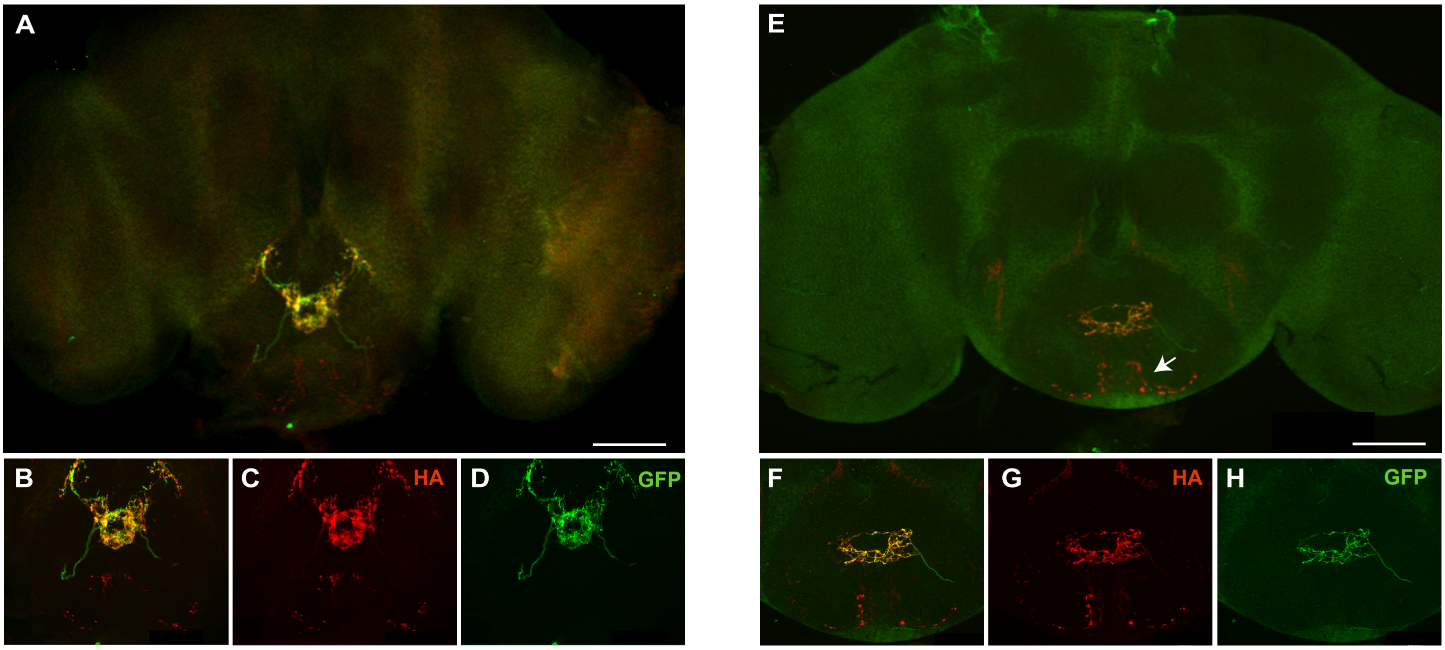

Supplement: Figure S3 — Gr22e and Gr59b neurons contact OA neurons in the suboesophageal ganglion. (A) GRASP-mediated GFP reconstitution specifically in the SOG is observed between Gr22e neurons expressing CD4::spGFP1-10 and synaptotagmin:hemagglutinin (UAS-syt:HA) (red, anti-HA) and OA neurons expressing CD4::spGFP11. GRASP reconstitution is detected by immunofluorescence using a monoclonal GFP antibody (green, Invitrogen, A-11120, Lot 764809). (B–D) Optical sections at higher magnification showing GRASP-mediated GFP reconstituted expression (D), syt:HA localization (C) and clear overlap or close association at synaptic-like puncta in the merged channel (B). (E) GRASP-mediated GFP reconstitution between Gr59b neurons expressing CD4::spGFP1-10 and UAS-syt:HA (red, anti-HA) and OA neurons expressing CD4::spGFP11. Regions in the SOG with only syt:HA expression are indicated (arrow) in addition to GFP-reconstitution contacts that show co-localization with syt-HA expression. (F–H) Higher magnification view of optical sections with GRASP-mediated GFP reconstitution (J), syt:HA localization (I), and the observed overlap in punctate patterns (H). Scale bars represent 20 µM. (PDF) [file pgen.1004356.s003.pdf]
