# Supplementary material for: Octopamine Neuromodulation Regulates Gr32a-Linked Aggression and Courtship Pathways in Drosophila Males
Source: PLoS Genet. 2014 May 22;10(5):e1004356. doi: 10.1371/journal.pgen.1004356 (PMC4031044; doi:10.1371/journal.pgen.1004356)

Figure S7 (Andrews et al.,). Quantification of aggression parameters in Gr32a-expressing and OA deficient males

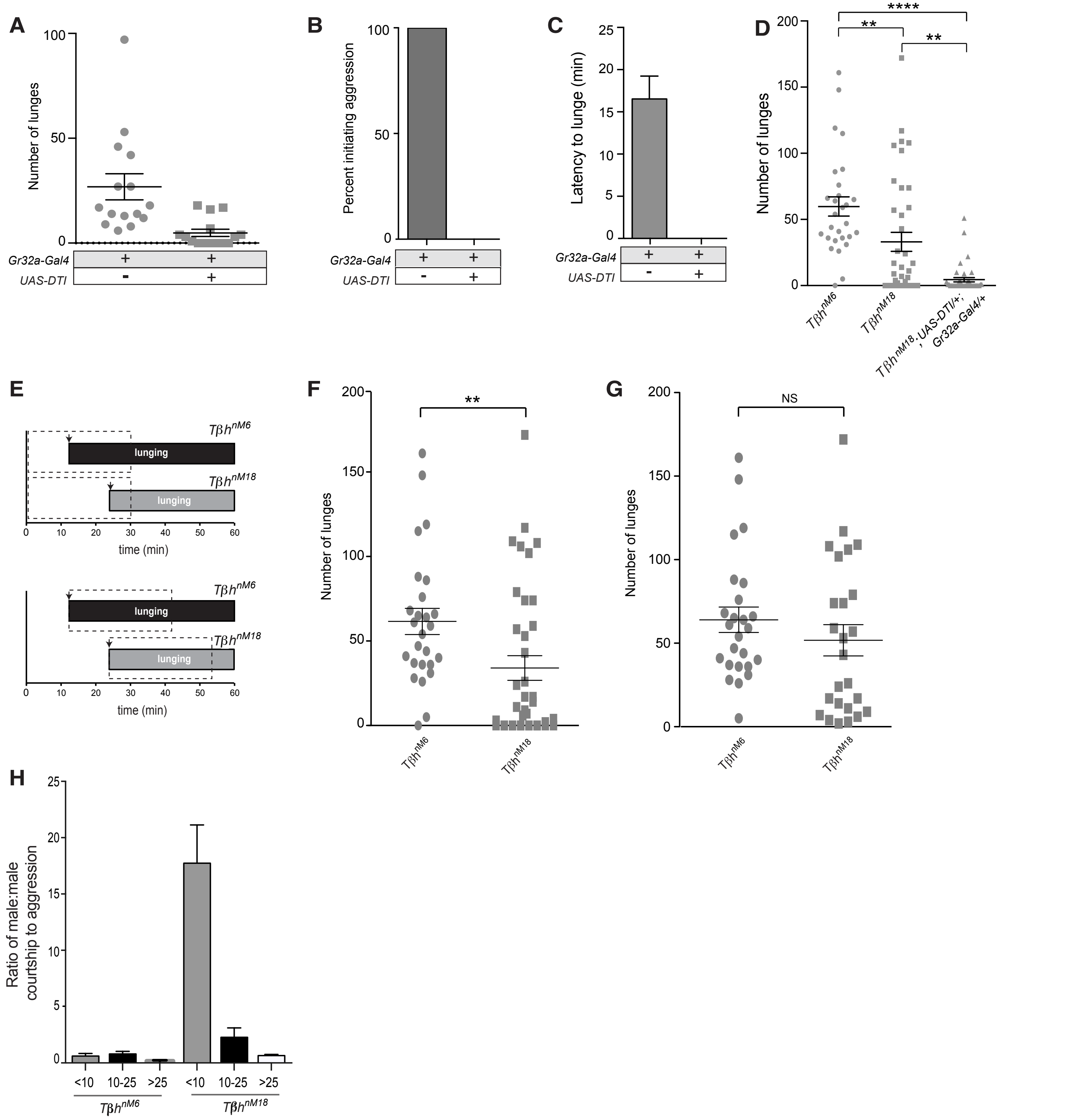

Supplement: Figure S7 — Defects in aggressive behavior parameters in Gr32a-expressing and OA deficient males. (A–C) Experimental males without Gr32a-expressing neurons (UAS-DTI;Gr32a-Gal4) do not exhibit aggressive behavior when paired with control males. (A) Males without Gr32a-expressing neurons display significantly fewer lunges than control males (+/Gr32a-Gal4). (B) Control males initiated aggression as measured by the first lunge in all assays, n = 15. (C) The latency to first lunge by control males is similar in pairings with experimental and control males (Figure 3). (D) The number of lunges by experimental tβhnM18;UAS-DTI/+; Gr32a-Gal4/+ males was significantly less than exhibited by control males (tβhM6) or males without OA (tβhnM18) (****p<0.0001, **p = 0.003). (E–G) Aggressive behavior or the component patterns that make up aggressive behavior are commonly quantified for a given period of time from the moment that pairs of flies are placed into a fight chamber (E, upper panel). This method of scoring does not take into account any substantial differences in the latency to begin fighting. Given the observed latency to initiate the fights, we quantified the number of lunges performed by each pair of males during a 30-minute period starting from the onset of aggression (lower panel). (F) If fights without lunges are scored as “zeros”, the numbers of lunges seen in fights between pairs of tβhnM18 males are significantly lower than the numbers seen in the genetic controls. One outlier value of 416 is observed in a tβhnM18 pairing. In this comparison with fights that do not exhibit fighting, tβhM6 and tβhnM18 are statistically different with the inclusion or absence of the outlying value (Mann-Whitney test, p value with outlier = 0.0049, p value without outlier = 0.0023) (G) When pairs that did not display lunges are excluded in the quantification, significant differences are not found in the lunge frequency between tβhnM18 and tβhM6 male pairs. One outlier value of 416 is observed in [file pgen.1004356.s007.pdf]
